# The Neural Substrate of Reward Anticipation in Health: A Meta-Analysis of fMRI Findings in the Monetary Incentive Delay Task

**DOI:** 10.1007/s11065-018-9385-5

**Published:** 2018-09-25

**Authors:** Robin Paul Wilson, Marco Colizzi, Matthijs Geert Bossong, Paul Allen, Matthew Kempton, MTAC, N. Abe, A. R. Barros-Loscertales, J. Bayer, A. Beck, J. Bjork, R. Boecker, J. C. Bustamante, J. S. Choi, S. Delmonte, D. Dillon, M. Figee, H. Garavan, C. Hagele, E. J. Hermans, ICCAM Consortium, Y. Ikeda, V. Kappel, C. Kaufmann, C. Lamm, S. E. Lammertz, Y. Li, A. Murphy, L. Nestor, M. Pecina, D. Pfabigan, D. Pizzagalli, L. Rademacher, R. Admon, T. Sommer, R. Stark, H. Suzuki, T. Van Amelsvoort, E. Van Hell, M. Vink, M. Votinov, D. Wotruba, Sagnik Bhattacharyya

**Affiliations:** 1 Department of Psychosis Studies, Institute of Psychiatry, Psychology and Neuroscience, King’s College London, De Crespigny Park, London, SE5 8AF UK; 2 Department of Psychiatry, Brain Center Rudolf Magnus, University Medical Center Utrecht, Utrecht, Netherlands; 3 Cognition, Neuroscience and Neuroimaging (CNNI) Laboratory, Department of Psychology, University of Roehampton, London, UK

**Keywords:** Monetary incentive delay task, Anticipation or reward, Healthy adults, fMRI, Meta-analysis

## Abstract

The monetary incentive delay task breaks down reward processing into discrete stages for fMRI analysis. Here we look at anticipation of monetary gain and loss contrasted with neutral anticipation. We meta-analysed data from 15 original whole-brain group maps (*n* = 346) and report extensive areas of relative activation and deactivation throughout the whole brain. For both anticipation of gain and loss we report robust activation of the striatum, activation of key nodes of the putative salience network, including anterior cingulate and anterior insula, and more complex patterns of activation and deactivation in the central executive and default networks. On between-group comparison, we found significantly greater relative deactivation in the left inferior frontal gyrus associated with incentive valence. This meta-analysis provides a robust whole-brain map of a reward anticipation network in the healthy human brain.

**Electronic supplementary material:**

The online version of this article (10.1007/s11065-018-9385-5) contains supplementary material, which is available to authorized users.

## Introduction

Reward processing in the brain is an iterative learning process involving goal-directed behaviour and adaptive decision-making in response to a stimulus. Stimulus presentation followed by receipt of reward increases the likelihood of a behaviour occurring again. A reward stimulus (incentive) has an intrinsic value (valence) which makes it salient, standing out from a background of stimulus bombardment. Incentives may be innately rewarding to an organism (e.g. sex, food), known as intrinsic rewards, or may be neutral at first and learnt by association, known as extrinsic rewards (e.g. money). The anticipation of a reward incentive prepares an approach behaviour, creating motivational salience, and the consumption of the reward reinforces motivational salience.

The neural mechanism of reward processing is beginning to be understood. Physiological work in primates revealed that reward prediction is mediated by dopaminergic neurons in the striatum (Schultz et al. 1997). Moreover a putative salience network has been identified by functional connectivity analysis (Seeley et al. 2007) which may be involved in choosing stimuli worthy of attention from a continuous stream of internally and externally generated inputs to the brain, thought to be anchored in the anterior cingulate and anterior insula (Uddin 2015).

**Fig. 1:**
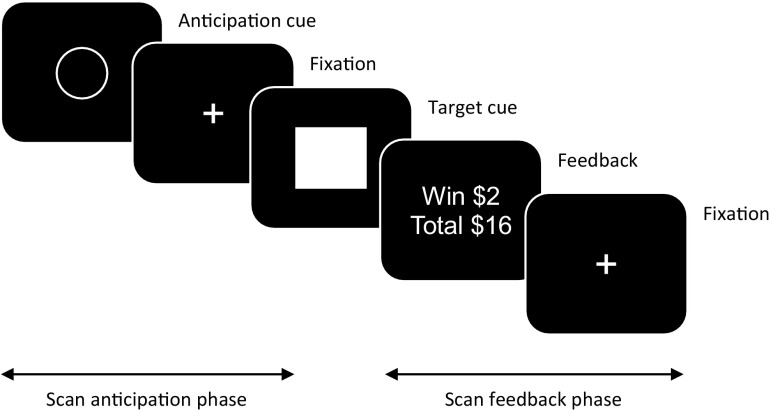
Example of visual cues presented during a trial of the monetary incentive delay task

The monetary incentive delay task is a widely used and validated reward processing task adapted for use in human fMRI studies to investigate motivational salience processes in health and disease. It was developed based on instrumental conditioning paradigms employed in animal studies (Schultz et al. 1997; Knutson et al. 2000). The monetary incentive delay task allows reward processing to be parsed into at least two distinct components, namely, ‘anticipation’ and ‘feedback’. Typically, the task consists of a sequence of three visual stimulus events (Fig. 1), (1) *anticipation,* a learned visual cue representing valence (e.g. financial gain-circle, loss-square, neutral-triangle) which elicits motivational salience, (2) *the target*, another learned visual cue (e.g. rectangle) to initiate the behaviour, usually pressing a button on time (a time-dependent motor task), and (3) *feedback* in the form of text or image indicating consummation (financial gain, loss, neutral) and dependent on performance.

When studied with fMRI, the two main phases when imaging data are acquired include (1) during and after the first visual stimulus (anticipation/motivational salience) and (2) during and after the third visual stimulus (feedback/consummation). Because of individual variation in performance, the duration of target presentation may be adjusted automatically within the program such that each participant experiences approximately the same success rates, usually set at around 66%. Typically, there are three main anticipation conditions, win, neutral or lose. The anticipation conditions are coupled with five main feedback conditions dependent on type of anticipation and performance (e.g. target hit or missed), namely, 1) anticipation-win-hit, 2) anticipation-win-miss, 3) anticipation-lose-hit, 4) anticipation-lose-miss and 5) anticipation-neutral. The neutral anticipation stimulus is used as a contrast to control for aspects of visual and motor processing commonly engaged during the different conditions. There are multiple variations that can be introduced, but in this meta-analysis we focus only on the anticipation conditions stimulating positive, neutral and negative salience through monetary gain or loss.

The original investigations of the monetary incentive delay task in healthy adults (Knutson et al. 2001; Knutson et al. 2000) reported that anticipation of reward (versus neutral) was associated with activation of multiple regions including areas implicated in both reward prediction (bilateral nucleus accumbens, bilateral caudate and left putamen) and the salience network (bilateral insula, right anterior cingulate gyrus).

Since the original studies, many more studies have used the monetary incentive delay task to investigate brain networks engaged in reward processing. The monetary incentive delay task has mainly been used to study differences between demographic groups (e.g. adolescents and adults, males and females), clinical populations (e.g. *major* depression, psychosis) or interventions (e.g. placebo). Several whole-brain meta-analyses have been conducted looking at reward anticipation in healthy adults using a variety of tasks and a mixture of different rewards, such as monetary, food, points, social feedback and pleasing images (Liu et al. 2011; Diekhof et al. 2012; Bartra et al. 2013). We identified only two meta-analyses focusing on reward anticipation in healthy adults using solely the monetary incentive delay task. The first meta-analysis of healthy adults using the monetary incentive delay task alone (Knutson et al. 2008) contrasted anticipation win directly with anticipation lose, whereas the second, more recent meta-analyses (Oldham et al. 2018) contrasted both win and lose with neutral conditions. Both analyses found that regions implicated in reward prediction and the salience network were activated.

All of the aforementioned meta-analyses, including the two focusing only on the reward processing in healthy adults employed the activation likelihood estimation technique (Turkeltaub et al. 2002) using published text coordinates. However, coordinate-based meta-analytic approaches cannot fully account for within study and random between study variation, because they do not include the full statistical images and exclude null findings unlike image-based meta-analyses (Müller et al. 2018). Coordinate-based methods such as activation likelihood estimation treat all foci of activation equally regardless of the strength of activation, and it has been shown that there is a poor similarity between coordinate and image based meta-analysis (Salimi-Khorshidi et al. 2009). The seed-based d mapping (SDM) meta-analytic technique (Radua et al. 2012) offers significant benefits over activation likelihood estimation, because it allows both thresholded coordinates and original group map image data to be combined creating maps effect-size. Furthermore, activation likelihood estimation and SDM answer slightly different questions. While the results of activation likelihood estimation-based meta-analysis may be interpreted as indicating the spatial convergence of previous findings, seed-based d mapping-based can be interpreted as direct increase or decrease in activity in the brain (Müller et al. 2018).

The primary objective of this study was to conduct a whole brain meta-analysis of fMRI studies employing only the monetary incentive delay task in healthy adults using the seed-based d mapping technique to investigate which brain regions are activated or deactivated during monetary reward anticipation. We specifically focused on the contrasts anticipation-win minus anticipation-neutral (AWAN) and anticipation-lose minus anticipation-neutral (ALAN), to confirm activation of regions implicated in reward processing, namely reward prediction and the salience networks.

## Methods

### Search Strategy and Study Selection

On 15/12/14 we searched the NICE Healthcare Database including EMBASE (Ovid), MEDLINE (Ovid), PsycINFO (Ovid) and CINAHL (EBSCO) using the terms ((“monetary” AND “incentive” AND “delay” AND “fmri”).ti,ab OR (“monetary” AND (“reward” OR “incentiv*” OR “anticipat*”) AND “fmri”).ti,ab) AND “article” [Limit to: Publication Year 2000–2014]. We complemented this with a cross-reference search of Pubmed on 16/12/14 with the general search term “monetary reward incentive anticipation fMRI”. Study inclusion criteria were (i) inclusion of healthy adults, (ii) used fMRI, (iii) used Monetary Incentive Delay Task, (iv) article available in English, (v) published in a peer-reviewed journal, (vi) conditions and contrasts of interest included, (vii) whole-brain analysis reported. We also included the placebo condition of intervention studies in healthy participants meeting criteria. Full articles were read and excluded if the above inclusion criteria were not met. The search was repeated using the same search terms and databases on 19/1/17, ranging from December 2014 onwards and excluding any repeated articles.

### Data Extraction

For all the articles that satisfied study inclusion criteria, all authors or corresponding authors were contacted by published email address in March 2015 requesting whole brain maps. Research groups who sent maps were included in the Monetary incentive delay Task Analysis Consortium (MTAC) (Supplementary References 1). If no maps were available for use, we manually extracted the whole-brain only coordinates from the published article for the conditions of interest for the healthy adult sample. For all articles (both maps-received and coordinates only), information on the MRI scanner, scanning sequence and timings, parameters of the monetary incentive delay task and performance data where available were extracted manually by two researchers (Colizzi and Wilson) and cross-checked for errors. This process of contacting authors was repeated for a follow-up literature search in March 2017. If no maps were available, we did not extract published article because of positive effect-size bias detected in the first round (see Results).

### Data Analysis: Seed-Based D Mapping

Meta-analysis was carried out using the seed-based d mapping software employing previously described methods (Radua et al. 2014; Radua and Mataix-Cols 2009; Radua et al. 2012, 2010). In brief, the aim of seed-based d mapping is to create voxel-level maps of effect-size measured as Hedge’s g allowing modelling of both positive and negative activations on the same map. For each reported peak coordinate and value (t, z or p), seed-based d mapping ensures surrounding voxels have a similar but smaller estimated effect size by multiplication with an un-normalised Gaussian kernel. If a voxel should have a value assigned from more than one coordinate, the values are averaged weighting by the square of the distance to each close peak. The data from each study are then weighted by the inverse of the sum of variance plus between study variance and combined using the random effects model DerSimonian-Laird estimator (DerSimonian and Laird 1986). This approach allows for studies with a larger sample size or lower variability to contribute more and creates a map of heterogeneity.

Manually extracted whole-brain coordinates and group maps were formatted for seed-based d mapping. For coordinates, this involved conversion to Tailarach, for group maps conversion to Neuroimaging Informatics Technology Initiative format, left-right correction and preprocessing including reslicing data into a common voxel size by interpolation (full width at half maximum = 20 mm). Meta-analysis produced mean maps with non-overlapping clusters of activation and deactivation for each contrast. A non-parametric approach was used where *p*-values and seed-based d mapping z-scores were created by randomisation, as opposed to standard z-scores.

In neuroimaging analyses, as well as in meta-analyses, the need for appropriate statistical thresholding to minimize the extent of false positive results must be balanced against the need for avoiding false negatives (Lieberman and Cunningham 2009). Typically, neuroimaging meta-analyses control for the false discovery rate to minimize false positive results, which we have also reported. However, it is worth noting that the choice of threshold is best guided by the specific research context (Müller et al. 2018). In the present study, we did not employ the conventional threshold, but instead combined an intensity threshold (*p* < 0.005) with a cluster extent threshold (cluster >10 voxels) that has been shown to result in acceptable Type II error rates (Lieberman and Cunningham 2009).

Thus seed-based d mapping generated mean maps were thresholded at the validated default settings p < 0.005, seed-based d mapping-z > 1.0, cluster>10 voxels. In seed-based d mapping, using a cluster size of 10 voxels and an uncorrected *p* = 0.005 has been shown empirically to be equivalent to corrected *p* = 0.05, optimally balancing sensitivity and specificity, and seed-based d mapping-z > 1 reduces the false positive rate (Radua et al. 2012). Given 100% parametric maps, this results in 100% sensitivity and a 3.5% false positive rate to produce the final whole brain maps of statistical significance, accompanied by an HTML document of main peaks, *p* value, seed-based d mapping-z-score, MNI coordinates, number of voxels per cluster and significant sub-peaks within each cluster (also with p value and seed-based d mapping-z-score). All results at the default seed-based d mapping-z > 1 threshold are reported in the supplementary material for the interested reader, however in the main text we have reported only those results that survived a more conservative threshold (z-score ≥ 5.0). The ‘5-sigma’ threshold for significance (Horton 2015) is a consensus agreement in other scientific disciplines, reflecting five standard deviations from the mean, or *p* < 0.0000003). We have adopted this threshold in order to further minimize the probability of detecting an effect by chance and to identify the most critical regions involved in motivational salience, reflecting high confidence that these are true positives when contrasting anticipation win or loss with neutral.

Jacknife sensitivity analysis of replicability, analysis of heterogeneity and publication bias are automated within the seed-based d mapping program. Jacknife analysis involves re-analysing mean maps multiple times by leaving out a single study each time. In order to interpret the many Jacknife mean maps, we thresholded for significance, binarized the data and combined into a single overlapping density map of significant data. This allowed visual inspection of areas of low density, signifying lower replicability across studies. Inter-study heterogeneity was calculated in which Q_H_ statistics are converted to standard z values to create a map. This map was overlaid on the final mean map for visual inspection of areas of overlapping significant heterogeneity with areas of thresholded activation or deactivation. Publication bias was estimated using standard funnel plots and Egger’s test for each reported peak. The funnel plots consisted of effect-size on the x-axis and standard error on the y-axis with bias tested using Egger’s test for asymmetry of the funnel plot.

The anatomical location of the peaks were identified using the Atlas of the Human Brain and associated BrainNavigator program (Mai et al. 2007). As the atlas did not cover the cerebellum, so the Talairach Daemon (Lancaster et al. 1997; Lancaster et al. 2000) was used, following MNI to Talairach conversion.

Following mean map analysis, the two contrasts AWAN and ALAN were compared using the automated linear model analysis which calculates the between-group difference based on statistical significance after Monte Carlo randomisation. In order to study potential confounders, automated linear model meta-regression was calculated, whereby the difference between the minimum and maximum values of regressors are returned with statistical significance based on Monte Carlo randomisation.

## Results

**Fig. 2:**
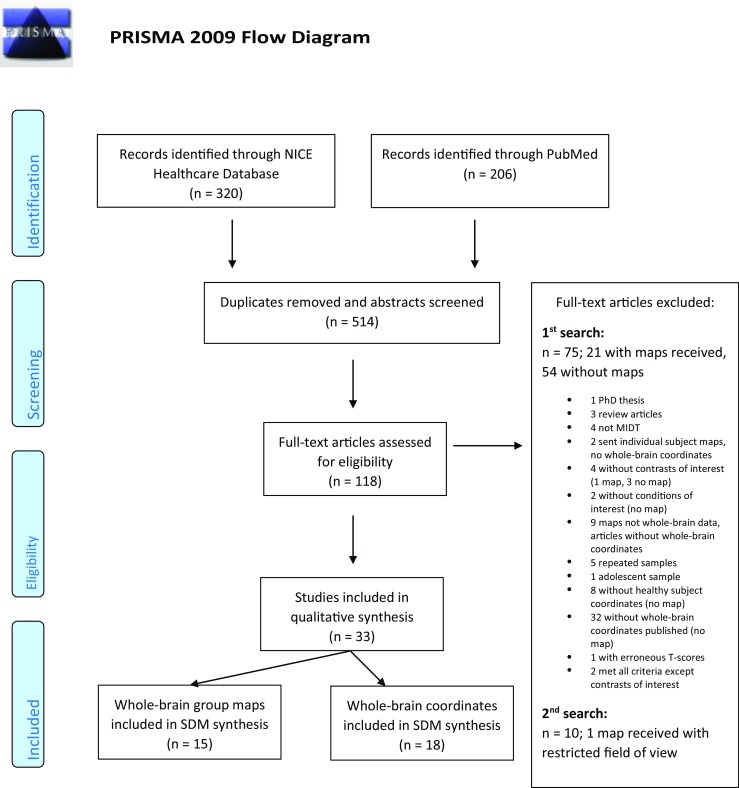
PRISMA flow diagram

Following removal of duplicates, and screening of abstracts, from an initial list of 288 articles a final selection of 108 articles were eligible for inclusion. We contacted all corresponding authors for the 108 articles of whom 70 responded. Brain maps corresponding to 36 articles were received. Of these, 21 were excluded for reasons detailed in the Prisma flow diagram (Fig. 2).

In total 15 sets of whole brain group maps were included in the meta-analysis of anticipation (Supplementary Table 1). Thirty-three articles were included in the first stage of the meta-analysis including 18 sets of whole-brain coordinates extracted from the text (Supplementary Table 1). No additional maps were included on repeating the literature search two years later. The omnibus sample size for all 33 non-overlapping samples (maps and text coordinates) was 728 healthy adults, mean age 30.3 years (SD 7.81), 60.6% male, and 98.8% right-handed (Table 1). See Supplementary Tables 1, 2 and 3 for full demographics, monetary incentive delay task specifics and fMRI acquisition and analysis for each study.

**Table 1:** Summary of available demographics for omnibus data of coordinates and group maps combined and of group maps only

contrast	n all datasets (text coordinates + group maps)	n sample size	pooled mean age (pooled SD^1^)	male %	Right handed %^2^	n group maps	n sample size	pooled mean age (pooled SD^1^)	male %	Right handed %^2^
AW-vs-AN	32	656	30.7 (8.15)	61.4	98.8	14	274	27.8 (6.09)	78	98.4
AL-vs-AN	21	494	31.6 (8.63)	64.8	99.3	11	246	27.4 (6.11)	71	97.8
TOTAL	33	728	30.3 (7.81)	60.6	98.8	15	346	27.4 (5.35)	76.5	98.4

^1^pooled SD excluding datasets where no SD reported; ^2^mean of available data, only 25/33 sets total: 24/32 AW, 21/21 AL

An initial omnibus analysis combined group maps and published text coordinates. However, we found significant bias for the two most significant peaks of the AWAN and ALAN contrasts (Supplementary Fig. 1, Supplementary Table 4). Given this finding and the theoretical discrepancies between coordinate and image-based meta-analysis previously discussed, we report only the *group map* meta-analysis from here on (Supplementary Table 5).

### Anticipation Win Minus Anticipation Neutral

This contrast was examined in a total sample size of 274 participants (Table 1).

#### Activation

**Fig. 3:**
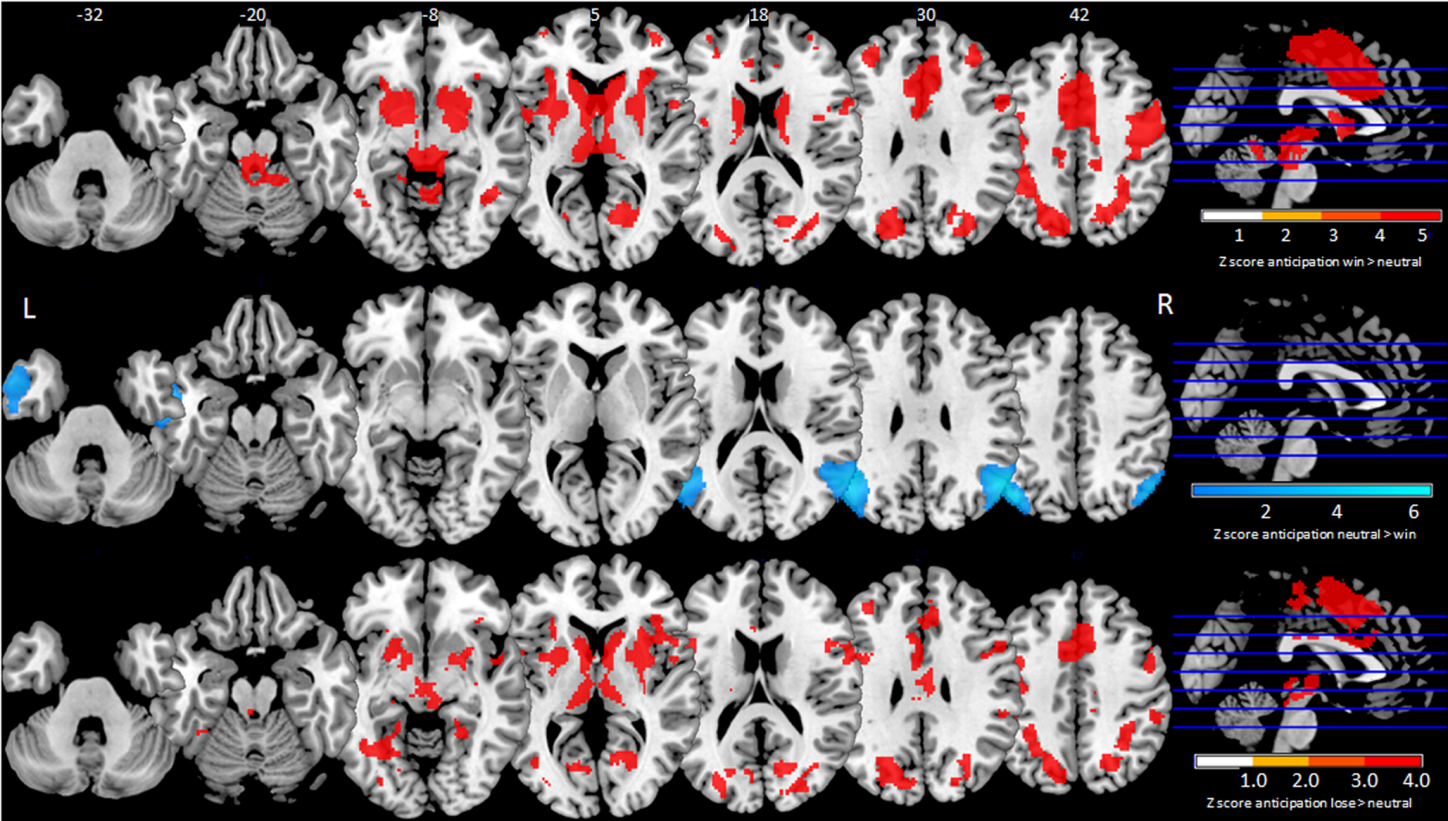
Mean maps thresholded to signed differential mapping z-score > 5. Top row - anticipation win-vs-anticipation neutral with activation in red. Middle row - anticipation win-vs-anticipation neutral with deactivation in blue. Bottom row - anticipation lose-vs-anticipation neutral with activation in red

Eleven main peaks in eleven clusters were identified (size: 10 to 25,114 voxels, z-score: 3.967 to 9.798, Table 2, Fig. 3). Eight main peaks and 144 subpeaks exceeded z = 5.0. The main peak of largest cluster was located in the right superior frontal gyrus lateral part extending widely to include, amongst 140 subpeaks, the caudate and putamen bilaterally, left fundus region of caudate (proximate to nucleus accumbens) and right nucleus accumbens. The remaining main peaks z > 5.0 were bilateral middle frontal gyri, bilateral inferior temporal gyri, left paracentral lobule, left occipital gyrus and left parahippocampal gyrus.

**Table 2:** Anticipation win-vs-anticipation neutral main peaks for SDM z-score > 5 or < −5

Peak MNI coordinate	SDM-z	P	FDR	Voxels	Anatomical Description	n subpeaks	Egger’s test p
Activation							
2,0,62	9.798	~0	>0.0001…	25,114	Right superior frontal gyrus, lateral part	169	0.910
34,40,26	6.424	~0	>0.0001…	405	Right middle frontal gyrus	9	0.919
-34,42,28	6.679	~0	>0.0001…	313	Left middle frontal gyrus	5	0.248
-16,-22,38	6.322	~0	>0.0001…	124	Left paracentral lobule	0	0.097
46,-52,-10	5.699	~0	>0.0001…	125	Right inferior temporal gyrus	2	0.139
-20,-72,10	5.476	~0	>0.0001…	83	Left occipital gyrus	1	0.123
-40,-56,-6	5.440	~0	>0.0001…	51	Left inferior temporal gyrus	0	0.354
-18,-42,-6	5.437	~0	>0.0001…	26	Left parahippocampal gyrus	0	0.910
Deactivation							
-52,-64,36	−6.434	~0	>0.0001…	4857	Left angular gyrus	12	0.670
54,-62,30	−5.253	~0	>0.0001…	1736	Right superior temporal gyrus	2	0.613

MNI (Montreal Neurological Institute), SDM-z (Signed Differential Mapping z-score), FDR (false discovery rate)

#### Deactivation

Thirteen main peaks in thirteen clusters were identified (size: 36–4857 voxels, z-score: 1.022–6.434, Table 2, Fig. 3). Two main peaks and one subpeak exceeded z = 5 located in the right superior temporal gyrus and left angular gyrus.

### Anticipation Lose Minus Anticipation Neutral

This contrast was examined in a combined sample of 246 participants (Table 1).

#### Activation

We found nineteen main peaks (z-score: 3.947–6.801, size: 12–11,315 voxels, Table 3, Fig. 3). Seven main peaks and 65 subpeaks exceeded z = 5.0 (Supplementary 4.3). The main peak of the largest cluster was located in the left superior frontal gyrus medial part extending widely to include, amongst 47 subpeaks, bilateral putamen and right caudate. In descending order, other main peaks z > 5 .0 were located in the left parieto-occipital transition zone, right supramarginal gyrus (2 main peaks), left hippocampus CA1, right fusiform gyrus and left middle frontal gyrus.

**Table 3:** Anticipation lose-vs-anticipation neutral main peaks for SDM z-score > 5 < −5

Peak MNI coordinate	SDM-z	P	FDR	Voxels	Anatomical Description	n subpeaks	Egger’s test p
Activation							
0,18,52	6.801	~0	>0.0001…	11,315	Left superior frontal gyrus medial part	131	0.731
-28,-70,26	5.753	~0	>0.0001…	1854	Left parieto-occipital transition zone	26	0.359
30,-48,38	5.352	~0	>0.0001…	1491	Right supramarginal gyrus	25	0.185
-30,-60,-10	6.286	~0	>0.0001…	461	Left hippocampus CA1	5	0.587
52,-34,36	5.207	~0	>0.0001…	174	Right supramarginal gyrus	0	0.654
24,-46,-8	5.28	~0	>0.0001…	88	Right fusiform gyrus	0	0.620
-36,36,32	5.056	~0	>0.0001…	70	Left middle frontal gyrus	0	0.757

MNI (Montreal Neurological Institute), SDM-z (Signed Differential Mapping z-score), FDR (false discovery rate)

#### Deactivation

No main peaks exceeded the raised threshold z > 5, though multiple peaks were significant (Supplementary Table 5.a-d for full breakdown).

### Between Group Comparison: AWAN Minus ALAN

**Fig. 4:**
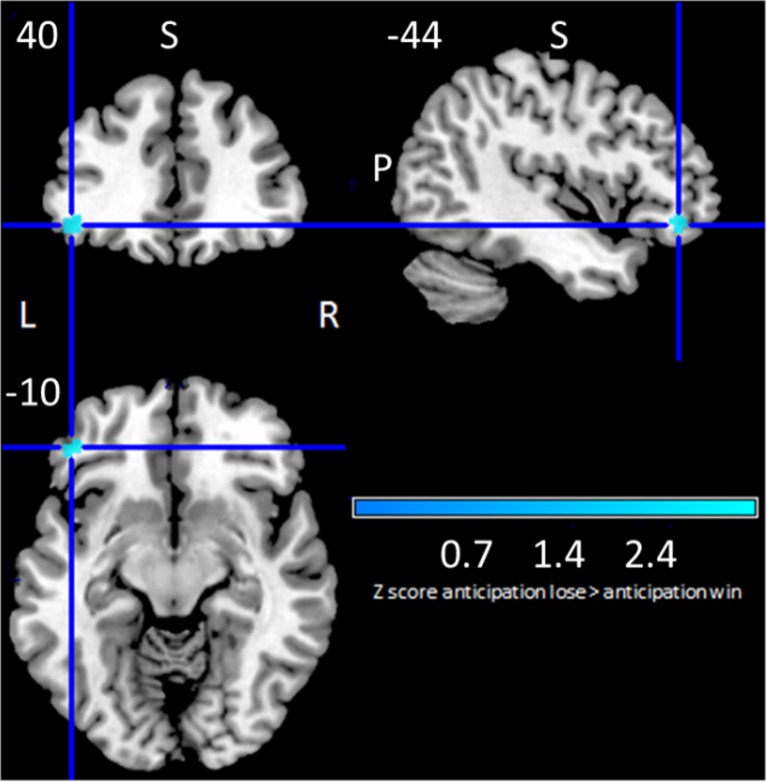
Between group comparison of anticipation win-vs-neutral minus anticipation lose-vs-neutral showing relative deactivation in blue

On between-group linear model comparison between AWAN and ALAN, we found significantly greater *deactivation* in AWAN compared to ALAN in the left inferior frontal gyrus opercular part (Fig. 4, Supplementary Table 6).

### Publication Bias, Heterogeneity, Sensitivity and Confounders

Funnel plots revealed no clear evidence of marked asymmetry on visual inspection for the higher thresholded main peaks for either contrast (Supplementary Fig. 1), also surviving Egger’s test for bias (Tables 2 & 3). Maps of significant heterogeneity (Q_H_) were overlaid on the mean maps for both contrasts to visually inspect for overlapping areas (Supplementary Fig. 2). For AWAN significant heterogeneity overlapped with activation in the bilateral ventral striatum and bilateral dorsal thalamic nuclei. For ALAN, overlap was seen in bilateral nucleus accumbens, bilateral medial superior frontal gyrus and the left precentral gyrus. Visual inspection of Jacknife analysis overlaid on the heterogeneity maps showed good replicability of all areas reported in both contrasts (Supplementary Fig. 3).

Outside of the task conditions, two independent regressors were available, namely, placebo intervention and scanner strength. Seventy of the 274 (26%) subjects in AWAN and 59 of the 246 (24%) in ALAN were given placebo (wash-out period 1 to 2 weeks). Overlaying heterogeneity maps for each contrast with meta-regression for placebo showed overlap with activation in AWAN in the bilateral ventral striatum, but no visual overlap in the ALAN contrast (Supplementary Fig. 4, Supplementary Table 8).

Regarding field strength, for AWAN 7 of 14 mean maps were generated using a 3 T scanner, for ALAN 5 of 11 maps were 3 T and the remainder for both 1.5 T. No areas of overlap in heterogeneity corresponded to areas of activation or deactivation in the mean maps (Supplementary Fig. 5, Supplementary Table 9).

### Discussion

The aim of the present study was to investigate which regions of the brain are robustly engaged by reward processing in healthy adult humans, specifically looking at motivational salience, that is, presentation of reward incentive and preparation of approach behaviour. Using all available fMRI group-map data for the anticipation of reward and loss conditions of the monetary incentive delay task in healthy adult humans, our meta-analysis shows that there are large areas of both *activation* and *deactivation* across the whole brain in motivational salience. For the first time, we report the pattern of deactivation in anticipation of reward in the monetary incentive delay task in healthy adult humans. We also report evidence of positive effect-size bias in the anterior cingulate (AWAN) and striatum (AWAN and ALAN) in the literature.

The results presented here are consistent with those reported in a recent activation likelihood estimation based meta-analysis of reward anticipation using the monetary incentive delay task in healthy adults (Oldham et al. 2018), but we report far more extensive areas of activation involving frontal, temporal, parietal and cerebellar regions. Furthermore, we have reported on areas of relative deactivation as well as publication bias, heterogeneity, replicability, and the potential effects of placebo and scanner strength.

We have confirmed a number of regions of activation reported in the original monetary incentive delay task studies of healthy adults (Knutson et al. 2001; Knutson et al. 2000) for AWAN, including bilateral insula (left insula originally reported as motor cortex), right nucleus accumbens, nucleus accumbens, bilateral caudate, left putamen, thalamus, right amygdala, right anterior cingulate gyrus (both reported as ‘right mesial prefrontal cortex’), right superior frontal gyrus medial part (reported as ‘right SMA’) and right cerebellum anterior lobe culmen (reported as cerebellar vermis). However, we did not find any activation in the right amygdala or left nucleus accumbens, an area of significant heterogeneity. We have also greatly extended the pattern of activity such that all previous unilateral peaks, except right amygdala, were found to be bilateral including putamen, superior frontal gyrus (medial and lateral), anterior cingulate gyrus, cerebellum anterior lobe culmen, and thalamic nuclei. We have provided greater resolution within the thalamus itself, finding activation in bilateral medial dorsal thalamic nuclei and two left ventral anterior thalamic nuclei. We report nine additional bilateral areas of activation including middle frontal gyri and inferior temporal gyri, precentral gyri, frontal operculae, paracingulate cortex, postcentral gyri, superior parietal lobules, precunei and insular gyri. We report twenty additional unilateral areas of activation (5 main peaks) including areas known to be implicated in both salience processing, such as the hippocampus (Crottaz-Herbette et al. 2005), and reward processing, such as the right parahippocampal gyrus and right inferior frontal gyrus (Brooks et al. 2013).

### Interpretation

As predicted, the striatum is strongly engaged in both anticipation of reward and loss, though not clearly differentiated when comparing the two contrasts. We found significant heterogeneity in this area in the anticipation of reward across all studies which may be explained in part by placebo effects.

Both reward and loss anticipation robustly engage key nodes of the salience network including anterior insular and anterior cingulate cortex. However, a mixed picture emerged for the anterior cingulate with anticipation of reward strongly activating bilateral anterior cingulate with a single main peak of deactivation in the left anterior cingulate. In contrast, anticipation of loss strongly activated the left anterior cingulate only with no detected deactivation. This pattern of activation may be reflected in the AWAL between-group comparison (Supplementary Table 6) showing a subthreshold (cluster size 8 voxels) difference in activation in the left anterior cingulate. The anterior insula was activated bilaterally in both contrasts. The insula cortex is considered a major cortical target of ascending interoceptive and visceromotor signals passing through thalamic nuclei (Uddin 2015) found to be functionally connected to amygdala, dorsomedial thalamus, hypothalamus peri-aqueductal grey matter (Seeley et al. 2007). We confirmed activity in these regions, though seemingly a different pattern for each contrast (AWAN - bilateral dorsomedial thalamic nuclei, right periaqueductal grey matter; ALAN - right posterior hypothalamic area, left basomedial nucleus of the amygdala).

The right anterior insular cortex, in particular, is also thought to be involved in switching between two other networks, namely, the central-executive and the default-mode networks (Sridharan et al. 2008). The central-executive network is considered the neural substrate underlying cognitive processes such as inhibition, interference control, working memory and cognitive flexibility (Diamond 2013) and is thought to be anchored in the dorsolateral prefrontal and lateral parietal cortex (Sridharan et al. 2008). We found strong bilateral activation of dorsolateral prefrontal cortex in both contrasts in the bilateral middle frontal gyri and bilateral superior frontal gyri, lateral part. Regarding the lateral parietal cortex we found significant but differing patterns of activation for each contrast. In both conditions, there was bilateral activation of the superior parietal lobule and unilateral activation of the right angular gyrus. However in AWAN there was *unilateral* activation of the left supramarginal gyrus and deactivation in the *left* angular gyrus, and in ALAN *bilateral* activation of the supramarginal gyrus and unilateral deactivation of the *right* angular gyrus.

The default-mode network is considered a state of cortical activity independent of external stimuli (originally considered to be ‘waking rest’) convergent with areas active in resting state fMRI (Andrews-Hanna et al. 2014) and task-induced deactivation (Andrews-Hanna, 2012). This network has been further subdivided into a ‘core’ anchored in the posterior cingulate cortex and anteromedial prefrontal cortex, as well as the ‘medial temporal’ and’ dorsal medial subsystems’ (Andrews-Hanna 2012). The core is associated with self-referential processes, the medial temporal subsystem corresponds to past and future autobiographical thought, episodic memory and contextual retrieval, and the dorsal medial subsystem corresponds to social cognition, story comprehension and semantic processing (Yeo et al. 2011). We found bilateral activation of the ‘core’ posterior cingulate in both anticipation contrasts. Regarding the medial temporal subsystem, we observed a differing pattern of activation in the ventromedial prefrontal cortex with unilateral activation of the right posterior orbital gyrus in AWAN and unilateral activation of the left intermediate orbital gyrus in ALAN. In AWAN in the medial temporal lobe, there was bilateral deactivation in the parahippocampal gyrus in conjunction with unilateral activation in the left parahippocampal gyrus. Yet in ALAN we reported unilateral activation of the left hippocampus CA1 region. With respect to the dorsomedial subsystem, on the one hand we found robust bilateral activation of the superior frontal gyrus, lateral part in both contrasts. On the other hand, we saw a mixed picture in the lateral temporal cortex. The inferior temporal gyrus was activated in both contrasts. In AWAN, there was activation in the right middle temporal gyrus, deactivation in the right middle gyrus and deactivation in the right superior temporal gyrus. In ALAN there was activation in the left middle temporal gyrus, deactivation in bilateral middle temporal gyri and deactivation in the left superior temporal gyrus.

The monetary incentive delay task is also a motor processing task and, as such, we observed broad bilateral activation of primary motor cortex (precentral gyrus), somatosensory cortex (post central gyrus), the supplementary motor area and multiple thalamic nuclei in both contrasts. The striatal region of the basal ganglia was activated, but no peaks were seen in the globus pallidus or subthalamic nuclei. Significant activity was also found in the cerebellum. In AWAN there was strong activation in bilateral anterior lobe of the cerebellum and a peak of deactivation in the right posterior lobe, but in ALAN a mixed picture of unilateral activation and deactivation in the right anterior lobe. It is known that there is an important function for the cerebellum in motivational salience, in keeping with a recent animal study (Cutando et al. 2013) suggesting a role in encoding expectation of reward, the growing understanding of the reciprocal connections between cerebellum and basal ganglia (Niendam et al. 2012) and cerebellar involvement in addiction (Moulton et al. 2014).

These observed patterns of activation are controlled by the neutral condition in which the only theoretical difference in task activation is the absence of motivational salience. Could it be that common areas of activation and deactivation are part of a motivational salience network, robustly engaging the striatum, salience network, parts of the central executive and default networks and associated motor regions? The picture emerging from the central executive and default network appears more complicated with robust activation in the posterior cingulate and dorsolateral prefrontal cortex, but a differing pattern emerging between anticipation of reward and loss in the lateral parietal cortex, ventromedial cortex, medial (including hippocampus) and lateral temporal cortex.

### Between-group comparisons

The first meta-analysis of healthy controls discussed earlier compared uncontrolled anticipation of reward directly with loss (Knutson and Greer 2008). We used a different approach (between-group linear model) comparing mean group maps for two controlled conditions, AWAN and ALAN. We report only one significant peak above threshold in the left inferior frontal gyrus operculum. This region was deactivated in both conditions, but significantly more deactivated in anticipating loss than reward. Additionally, the left anterior cingulate gyrus was active in both contrasts, significantly more so anticipating reward than while anticipating loss, but just under threshold (Supplementary Table 6). The left inferior frontal gyrus has previously been implicated in response inhibition (Swick et al. 2008; Bhattacharyya et al. 2015) and the attribution of aspects of stimulus salience and attentional allocation of resources (Seeley et al. 2007; Bhattacharyya et al. 2012; Downar et al. 2002). Might this left inferior frontal activation reflect differential allocation of cognitive resources according to motivational salience or could this directly reflect a representation of valence?

### Limitations

Our findings are limited by the reported sample demographics, constraints of the monetary incentive delay task, the data acquisition and analysis, the methods of meta-analysis and the brain atlas used. Across all the studies, we were only able to report sample size, age, gender and handedness. Other variables such as substance use, education, IQ, socioeconomic status and ethnicity were too inconsistently reported for any meaningful interpretation.

In terms of the monetary incentive delay task, there was significant variation across designs. The maps we received were from many different international locations including Europe, the USA, Japan and South Korea. Therefore the financial incentive could have different meanings to different samples, although superficially they appear to be similarly small amounts of money. There was inconsistent reporting of, and variation in, the duration of all the phases of each trial, including monetary incentive stimulus presentation, anticipation, target, feedback and inter-stimulus interval. The data are gathered over multiple trials varying from 44 to 180 in each study, and the target hit rate was either unreported or varied from 50% to 75% which could have influenced any neural substrate for the temporally bound reward prediction error signal. The monetary incentive delay task is not designed to capture temporal learning or any reward prediction error signal. Variants of the monetary incentive delay task have been developed that introduce contingency, varying the predictability of reward receipt following action, and we have included a single group map in the AWAN condition introducing this (Li et al. 2014). Lastly, overall performance data was not consistently reported, preventing meaningful behavioural interpretation.

Regarding data acquisition and analysis, a variety of scanners and sequences were used in each study. There was variation in acquisition and echo time, number and thickness of slices, resolution and analysis software. All of these are documented in Supplementary Table 3 for reference. Only two potential confounders were available for statistical analysis, scanner strength and placebo condition, though meta-regression and comparison with heterogeneity and mean maps did not suggest any significant effects on our results.

The most important limitation to our method was the exclusion of over half of the studies. We felt this to be an essential step after identification of the positive effect-size bias in published coordinate-based data. The main peaks of the omnibus analysis (text coordinates and group maps) are reported in Supplementary Table 4 for reference. Secondly, because we found such a large number of significant peaks, we could not report all the data generated by this meta-analysis and applied a very conservative threshold. Subsequently, on the one hand, we feel our results are extremely robust, but on the other, many regions which may be of interest are not discussed. However, all these data are recorded in the Supplementary Table 5.

In terms of the between-group linear model comparison of AWAN and ALAN, there was a difference in gender proportion between AWAN (78%) and ALAN (71%), though this effect just fails to achieve nominal significance (χ2 (1) = 3.34, *p* = 0.07). However, 10 of the 15 studies (*n* = 194) for which we received group maps, sent both AWAN and ALAN contrasts, such that the majority of the data was repeated-measures within-group, strengthening the results.

Finally, the brain atlas used for anatomical location, while strong on detail, is based on the dissection of a single 24 year old male brain and does not cover the cerebellum necessitating additional use of the Tailarach Daemon.

Notwithstanding these limitations, this image-based meta-analysis uses data from a large sample of healthy individuals and reveals robust engagement of brain areas that have previously been linked to the anticipation of reward as well as novel brain areas, providing a definitive map of the reward anticipation network in the healthy human brain.

## Electronic supplementary material

ESM 1 (DOCX 4180 kb)
