# Supplementary material for: The Neural Substrate of Reward Anticipation in Health: A Meta-Analysis of fMRI Findings in the Monetary Incentive Delay Task
Source: Neuropsychol Rev. 2018 Sep 25;28(4):496–506. doi: 10.1007/s11065-018-9385-5 (PMC6327084; doi:10.1007/s11065-018-9385-5)
Supplement: Supplementary file 1 — (DOCX 4180 kb) [file 11065_2018_9385_MOESM1_ESM.docx]

Supplementary Materials

**Supplementary References 1: Monetary Incentive Delay Task Analysis Consortium (MTAC) listed in alphabetical order**

Abe, N, Barros-Loscertales, AR (Costumero et al. 2013), Bayer, J, Beck, A, Bjork, J, Boecker, R, Bustamante, JC (Bustamante et al. 2014), Choi, JS (Choi et al. 2012), Delmonte, S, Dillon, D, Figee, M, Gallagher, L, Garavan, H (Nestor et al. 2010), Hagele, C, Hermans, EJ, ICCAM Consortium, Ikeda, Y (Saji et al. 2013, Funayama et al. 2014), Kappel, V (Kappel et al. 2014), Kaufmann, C (Kaufmann et al. 2013), Lamm, C (Pfabigan et al. 2014), Lammertz, SE (Pfabigan et al. 2014), Li, Y (Li et al. 2014), Murphy, A, Nestor, L (Nestor et al. 2010), Pecina, M (Pecina et al. 2014), Pfabigan, D (Pfabigan et al. 2014), Pizzagalli, D, Rademacher L, Roee, A, Stark, R, Suzuki, H (Saji et al. 2013, Funayama et al. 2014), Van Amselvoort, T (da Silva Alves et al. 2011), Van Hell, E (van Hell et al. 2012, Jansma et al. 2013), Vink M (de Leeuw et al. 2015), Votinov, M, Wotruba, D

**Supplementary Table 1. Available demographics for all 33 studies included in omnibus analysis including coordinate-based and group map data sources**

| Author | Country | Data(maps or coordinates) | n | Age (SD) | Male % | Right-handed % | Smoking | Alcohol | Drugs | Years education | FSIQ | SES | Ethnicity |
| --- | --- | --- | --- | --- | --- | --- | --- | --- | --- | --- | --- | --- | --- |
| De Leeuw (de Leeuw et al. 2015) | Netherlands | Maps | 29 | 30.3 (1.7) | 41 | 100 | 1/29 | n/a | 3 CU last12/12 | n/a | 117 (3) | n/a | n/a |
| Bustamante (Bustamante et al. 2014) | Spain | Maps | 18 | 37.4 (8.2) | 100 | 100 | n/a | n/a | 0 | 9.56 (2.2) | n/a | n/a | n/a |
| Funayama (Funayama et al. 2014) | Japan | Maps | 20 | 29.9 (n/a) | 60 | 100 | n/a | n/a | n/a | n/a | n/a | n/a | n/a |
| Kappel (Kappel et al. 2014) | Germany | Maps | 20 | 23.7 (3.4) | 100 | 100 | 6/20 | 304.3g/month ± 284.2 | n/a | 12.1 (1.4) | 108 (11.3) | 5.55 | n/a |
| Li (Li et al. 2014) | France | Maps | 20 | 31 (7.3) | 100 | 100 | 0.1 (0.3) Fagerstrom test nicotine dependence | 4.2 (3.5)  Alcohol use disorders identification test | n/a | 13.2 (1.7) | n/a | n/a | n/a |
| Pecina (Pecina et al. 2014) | USA | Maps | 72 | 26 (4.24*) | 53 | n/a | n/a | <5 drinks/week | 0 | n/a | n/a | n/a | 75% Caucasian, 18% African American |
| Pfabigan (Pfabigan et al. 2014) | Austria | Maps | 25 | 23.8 (3.6) | 48 | 100 | n/a | n/a | n/a | n/a | n/a | n/a | n/a |
| Costumero (Costumero et al. 2013) | Spain | Maps | 44 | 23.4 (4.1) | 100 | 98 | n/a | n/a | 0 | 13.8 (22) | n/a | n/a | n/a |
| Jansma (Jansma et al. 2013) | Netherlands | Maps | 11 | 21.2 (2.65*) | 100 | n/a | 0.06 cigs/day | 13.2u/week | 22.6 times cannabis used in last year, other illicit 0.73 | n/a | 105 (1.5) | n/a | n/a |
| Kaufmann (Kaufmann et al. 2013) | Germany | Maps | 19 | 34.9 (11.8) | 42 | 84 | n/a | n/a | 0 last 3/12 | n/a | 107 (12) | n/a. | n/a |
| Saji (Saji et al. 2013) | Japan | Maps | 18 | 29.6 (6.94) | 56 | 100 | n/a | n/a | n/a | n/a | n/a | n/a | n/a |
| Choi (Choi et al. 2012) | Korea | Maps | 15 | 26.6 (4.29) | 100 | 100 | 9.3 cigs/day (8 smokers) | 0 | 0 | 14.27 (1.4) | 114 (7.1) | n/a | n/a |
| Van Hell (van Hell et al. 2012) | Netherlands | Maps | 11 | 21.7 (2.3) | 100 | n/a | 3 cigs/week (SD 8.4, 0-28) | 16.7 U/week (SD 8.7, 2-40) | CU 17.9 times last 12/12 (SD 13.4, 5-52), other SU 1.3 /lifetime (SD 1.6, 0-4) | n/a | 105 (6) | n/a | n/a |
| Da Silva Alves (da Silva Alves et al. 2011) | Netherlands | Maps | 10 | 35.8 (10.4) | 100 | 100 | n/a | 0 | 0 | 14.8 (2.3) | n/a | n/a | n/a |
| Nestor (Nestor et al. 2010) | Ireland | Maps | 14 | 23.1 (4.49*) | 79 | 100 | 10 cigs/day (SD 2.4), 15/30 days used (SD 4.2) | 7.8 (SD 1.4) | CU 3 lifetime joints (SD 0.6); hallucinogenic x2 lifetime (SD 0.4); MDMA 3.6 lifetime use (SD 0.9); cocaine 4.8 lifetime (SD 0.3); amphetamine 3.3 lifetime (SD 1.2) | 16.1 (0.4) | 123 (0.8) | n/a | n/a |
| Damiano (Damiano et al. 2014) | USA | Coords | 31 | 23.58 (3.15) | 45 | 100 | n/a | n/a | n/a | n/a | 109 (2.46) | n/a | Non-Hispanic Caucasian, USA |
| Balodis (Balodis et al. 2012) | USA | Coords | 14 | 37.1 (11.3) | 71 | 93 | 2 current smokers | no past alcohol abuse/dependence | monitored prescan | n/a | 107 (13.2) | n/a | n/a |
| Enzi (Enzi et al. 2012) | Germany | Coords | 15 | 34.7 (8.3) | 53 | 100 | n/a | n/a | n/a | 14.94 (1.9) | n/a | n/a | n/a |
| Wu (Wu et al. 2014) | USA | Coords | 49 | 50 (16.5) | 47 | 106 | n/a | n/a | n/a | n/a | n/a | n/a | n/a |
| Kirk (Kirk et al. 2014) | USA | Coords | 44 | 36.5 (9.7) | 45 | n/a | n/a | n/a | n/a | 15.2 (1.7) | n/a | 2.7 (see note) | n/a |
| Romanczuk-Seiferth (Romanczuk-Seiferth et al. 2014) | Germany | Coords | 17 | 37.41 (11.76) | 100 | n/a | 8.03±8.30 cigarettes/day | 134.50±151.51 grams of alcohol 7 month | no drug dependence | 11.35 (1.5) | n/a | n/a | n/a |
| Weiland (Weiland et al. 2014) | USA | Coords | 12 | 30.9 (9) | 0 | 100 | "non-smoking" |  | no substance abuse/dependence, exposure last 6/12 to CNS prescription or illicit drug | n/a | n/a | n/a | n/a |
| Treadway (Treadway et al. 2013) | USA | Coords | 38 | 22 (4**) | 53 | n/a | n/a | n/a | n/a | n/a | n/a | n/a | n/a |
| Vaidya (Vaidya et al. 2013) | USA | Coords | 18 | 27.72 (1.36) | 50 | 100 | n/a | n/a | n/a | n/a | n/a | n/a | n/a |
| Ossewaarde (L. Ossewaarde et al. 2011) | Netherlands | Coords | 19 | 24.6 (7.1) | 47 | n/a | n/a | n/a | monitored prescan | n/a | n/a | n/a | n/a |
| Ossewaarde (Lindsey Ossewaarde et al. 2011) | Netherlands | Coords | 13 | 20 (1.8) | 0 | 100 | n/a | n/a | n/a | n/a | n/a | n/a | n/a |
| Beck (Beck et al. 2009) | Germany | Coords | 19 | 41.68 (8.97) | 100 | 100 | 9.68 cigs/day (sd8.65) | 4.06kg pure alcohol/year (SD 4.27) | n/a | 11.65 (1.7) | 108 (7.91) | 6.06 | n/a |
| Knutson (Knutson et al. 2003) | USA | Coords | 12 | 31 (n/a) | 50 | 100 | n/a | n/a | n/a | n/a | n/a | n/a | n/a |
| De Greck (de Greck et al. 2011) | Germany | Coords | 20 | 37 (10.6) | 40 | 80 | n/a | n/a | n/a | n/a | n/a | n/a | n/a |
| Spreckelmeyer (Spreckelmeyer et al. 2009) | Germany | Coords | 32 | 28.5 (7.25**) | 50 | 100 | "non-smoking" | n/a | n/a | n/a | n/a | n/a | n/a |
| Knutson (Knutson et al. 2008) | USA | Coords | 12 | 28.67 (4.25) | 33 | n/a | n/a | No alcohol abuse last 6/12, no Hx alcohol dependence | no substance abuse last 6/12, no Hx substance dependence | n/a | n/a | n/a | n/a |
| Knutson (Knutson et al. 2001b) | USA | Coords | 9 | 26.45 (5.85) | 22 | 100 | n/a | n/a | n/a | n/a | n/a | n/a | n/a |
| Knutson (Knutson et al. 2001a) | USA | Coords | 8 | 31 (n/a) | 50 | 100 | n/a | n/a | n/a | n/a | n/a | n/a | n/a |

SD (standard deviation), FSIQ (full scale intelligence quotient), SES (socioeconomic status), CU (cannabis use)

**Supplementary Table 2: Monetary incentive delay task parameters for all 33 studies included in omnibus analysis including coordinate-based and group map data sources**

| Author | monetary incentive | Placebo | Trial duration /ms | No. trials | Task duration /s | t stimulus /ms | t anticipation /ms | t target /ms | t delay /ms | t feedback /ms | t inter stimulus interval /ms | Planned hit rate % | Contrasts (-vs-neutal) |
| --- | --- | --- | --- | --- | --- | --- | --- | --- | --- | --- | --- | --- | --- |
| De Leeuw (de Leeuw et al. 2015) | €1 | N | 9571 | 60 | 575 | 750 | 779-6729 | . | . | . | 1029-6979 | 50 | AW, FWH, FWM |
| Bustamante (Bustamante et al. 2014) | €0.2, 3 | N | 8000 | 120 | 960 | 500 | 2000-2250 | 100 | 2000-4000 | 1500 | 2000-4000 | 75 | AW, AL, |
| Funayama (Funayama et al. 2014) | ¥20, 100, 500 | Y (2 week washout) | . | 180 | . | 2000 | 2000-2500 | 160-470 | 1130-1940 | 1900 | . | 66 | AW, AL, FWH, FWM, FLH, FLM |
| Kappel (Kappel et al. 2014) | *0.2, 1, 5 | N | 6000 | 144 | 1320 | 250 | 2000-2500 | 160-260 | . | 1650 | . | 66 | AW |
| Li (Li et al. 2014)* | €1,2,3,10,11,12 | N | . | 171 | . | 2500 | 1500-4500 | 1000 | . | 1500 | 2000-5000 | . | AW, FWH, FWM |
| Pecina (Pecina et al. 2014) | USD $0.2, 1, 5 | N | 6000 | 144 | . | . | . | . | . | . | . | . | AL |
| Pfabigan (Pfabigan et al. 2014) | €2 | N | . | 100 | . | 1000 | 2000-2500 | 264+/- |  | 1000 | 3000-7000 | 50 | AW, AL |
| Costumero (Costumero et al. 2013) | €0.2, 3 | N | . | 120 | 960 | 500 | 2000-2250 | 100 | 2000-4000 | 1500 | 2000-4000 | . | AW, AL |
| Jansma (Jansma et al. 2013) | €0.1, 5 | Y (2 week washout) | . | 44 | 711 | 500 | 4300-10300 | 280+/- | . | 1000 | 1000-21000 | 50 | AWS, AL, |
| Kaufmann (Kaufmann et al. 2013) | €0.1, 0.6, 3 | N | 11600 | 144 | 696 | 250 | 3740-4240 | 150-500 | 1420-1720 | 1870 | 3280-3780 | 66 | AW, AL, FWH, FWM, FLH, FLM |
| Saji (Saji et al. 2013) | ¥0, 100, 300 | Y (1 week washout) | 4370 | 144 | 780 | 250 | 2000-2500 | 160-260 | . | 1650 | 1000 | 66 | AW, AL |
| Choi (Choi et al. 2012) | ₩1000 | N | . | . | 1076.4 | 350 | 4180-4480 | 200-500 | . | 1500 | 5170-9850 | . | AW, AL |
| Van Hell (van Hell et al. 2012) | €0, 2 | Y (2 week washout) | 8000 | 48 | . | . | 4300-10300 | . | . | . | 0-30000 | 50 | AW, FWH |
| Da Silva Alves (da Silva Alves et al. 2011) | €0.2, 1, 5 | Y (8 day washout | 6000 | 144 | 6000 | 250 | 2000-2500 | 160-260 | . | 1650 | 5000 | 66 | AW, AL |
| Nestor (Nestor et al. 2010) | €0, 0.5 | N | 6000-18000 | 81 | 1020 | 2000-8000  (stimulus merged with anticipation) | | 400 | . | 1500 | 2000-8000 | 50 | AW, AL, FWH, FWM, FLH, FLM |
| Damiano (Damiano et al. 2014) | USD $1 | N | 12000 | 80 | . | 2000 | 2000-2500 | 500 | . | 3000 | . | 67 | AW |
| Balodis (Balodis et al. 2012) | USD $1, 5 | N | 12000 | 110 | 1200 | 1000 | 3000-5000 | . | 4000-6000 | 1200 | . | 66 | AW, AL |
| Enzi (Enzi et al. 2012) | €0.1, 0.6, 3 | N | . | 90 | . | 250 | 3740-4240 | 160-360 | 1500-2200 | 1650 | 4000 | 67 | AW, AL |
| Wu (Wu et al. 2014) | USD $0.50, 5 | N | 10000 | 90 | . | 2000 | 2000-2500 | 150-500 | . | 2000 | 2000-6000 | 66 | AW, AL |
| Kirk (Kirk et al. 2014) | USD $0, 1, 5 | N | 2500 | 96 | . | 2500 | 2000-2500 | 160-260 | 500 | 2000 | 1000-1500 | 66 | AW, AL |
| Romanczuk-Seiferth (Romanczuk-Seiferth et al. 2014) | €1 | N | 9500 | 75 | 712.5 | 500 | 2000-4500 | 150-500 | 1000-3000 | 1500 | 4000-7000 | 67 | AW, AL |
| Weiland (Weiland et al. 2014) | USD $0.20, 1, 5 | N | 6000 | 72 | 2x300 | 2000 | 2000 | 200-300 | 1700-1800 | . | . | 66 | AW |
| Treadway (Treadway et al. 2013) | USD $0.2, 1, 5 | N | . | 180 | 4x464 | 1000 | 2000-2500 | 160-260 | . | 1650 | . | 66 | AW, AL, FWH, FLM |
| Vaidya (Vaidya et al. 2013) | USD $.20, $1 and $5.00 |  | . | 162 | 3x600 | . | . | . | . | . | 4000 | 66 | AW |
| Ossewaarde (L. Ossewaarde et al. 2011) | €1 | N | 10000 (mean) | 50 | 750 | 3500-8500  (stimulus merged with anticipation) | | 150-500 (+10ms if previous miss, -20ms if previous hit)) | 1200-5300 | 500 | . | 33 | AW |
| Ossewaarde (Lindsey Ossewaarde et al. 2011) | €1 | N | 10000 (mean) | 50 | 750 | 3500-8500  (stimulus merged with anticipation) | | 150-500 (+10ms if previous miss, -20ms if previous hit)) | 1200-5300 | 500 | . | 33 | AW |
| Beck (Beck et al. 2009) | €0.1, 0.6, 3 | N | 7690 | 144 | 840 | 250 | 2250-2750 | 200-1000 | . | 1650 | 3530 | 67 | AW, AL |
| Knutson (Knutson et al. 2003) | USD $0.20, 1, 5 | N | 6000 | 144 | 2x432 | 250 | 2000-2500 | 160-260 | . | 1650 | . | 66 | AW, AL |
| De Greck (de Greck et al. 2011) | €1 | N | . | 180 | 1890 | 250 | 2250-2750 | 200-500 | . | 1650 | 4000-5000 | 66 | AW |
| Spreckelmeyer (Spreckelmeyer et al. 2009) | €0.20, 1, 3 | N | . | 88 | 840 | 240 | 2250-2750 | 160-260 | . | 1650 | 2500-5000 | 66 | AW |
| Knutson (Knutson et al. 2008) | USD $0.20, 1, 5 | N | 6000 | 180 | 1080 | 250 | 2000-2500 | 160-360 | . | 1650 | . | 66 | AW, AL |
| Knutson (Knutson et al. 2001b) | USD $1 | N | 6000 | 54 | 324 | 250 | 2000-2500 | 160-260 | . | 1650 | . | 66 | AW |
| Knutson (Knutson et al. 2001a) | USD $0.20, 1, 5 | N | 6000 | 144 | 2x600 | 250 | 2000-2500 | 160-260 | . | 1650 | . | 66 | AW, AL |

* uncertainty included in anticipation (probability of reward if successful 25, 75, 100%, and ‘low’ or ‘high’ intensity monetary reward), reaction to target based on visual discrimination within fixed time, not speed reaction time; AW (anticipation win), AL (anticipation lose), FWH (feedback win hit), FWM (feedback win miss), FLH (feedback lose hit), FLM (feedback lose miss)

**Supplementary Table 3: fMRI data acquisition and analysis parameters for all 33 studies included in omnibus analysis including coordinate-based and group map data sources**

|  | **Scanner** | **Sequence** | **Strength /Tesla** | **TR /ms** | **TE /ms** | **Software** | **FWHM /mm** | **No. slices** | **Slice thickness /mm** | **Resolution (resampled)** | **Corrected** | **Threshold** |
| --- | --- | --- | --- | --- | --- | --- | --- | --- | --- | --- | --- | --- |
| De Leeuw (de Leeuw et al. 2015) | Philips Achieva | 2D EPI-SENSE (sensitivity encoding) | 3 | 1600 | 23 | SPM5 | 8 | 30 | . | 4mm isotropic voxel | n/a | n/a |
| Bustamante (Bustamante et al. 2014) | Siemens Avanto | T2 * gradient | 1.5 | 2000 | 30 | SPM8 | 8 | 30 | 3.5 | (3x3x3mm) | n/a | n/a |
| Funayama (Funayama et al. 2014) | Phillips Electronics Intera Achieva Nova | GE-EPI | 1.5 | 2000 | 40 | SPM8 | 8 | 28 | 5 | (2x2x2mm) | n/a | n/a |
| Kappel (Kappel et al. 2014) | GE Signa | T2*-weighted in-/out-spiral pulse | 3 | 2300 | 27 | SPM8 | . | 29 | . | . | n/a | n/a |
| Li (Li et al. 2014) | Siemens Sonata | GE-EPI | 1.5 | 2500 | 60 | SPM2 | 10 | 26 | . | 3.4x3.4x4mm | n/a | n/a |
| Pecina (Pecina et al. 2014) | General Electric Signa | T2* weighted pulse sequence (single-shot combined spiral in/out, GE) | 3 | 2000 | 30 | SPM8 | 6 | 29 | 4 | . | n/a | n/a |
| Pfabigan (Pfabigan et al. 2014) | Siemens TIMTrio | Gradient-recalled EPI-sequence with distortion correction | 3 | 1800 | 38 | SPM8 | 8 | 23 | . | 1.5x1.5x3mm | n/a | n/a |
| Costumero (Costumero et al. 2013) | Siemens Avanto | EPI | 1.5 | 2000 | 30 | SPM5 | 6 | 30 | 3.5 | (3mm^3^) | n/a | n/a |
| Jansma (Jansma et al. 2013) | Philips Achieva | SENSE PRESTO | 3 | 22.5 | 33.2 | SPM5 | 8 | 40 | . | 4mm isotropic voxel | n/a | n/a |
| Kaufmann (Kaufmann et al. 2013) | Siemens Sonata | GE-EPI | 1.5 | 1870 | 40 | SPM8 | 8 | 33 | . | 3x3x3.5mm | n/a | n/a |
| Saji (Saji et al. 2013) | Phillips Electronics Intera Achieva Nova | GE-EPI | 1.5 | 2000 | 40 | SPM8 | 8 | 28 | 5 | (2x2x2mm) | n/a | n/a |
| Choi (Choi et al. 2012) | Siemens Avanto | GE-EPI | 1.5 | 2340 | 52 | SPM8 | 4 | 25 | . | (3x3x3mm) | n/a | n/a |
| Van Hell (van Hell et al. 2012) | Philips Achieva with Quasar dual gradient set | SENSE-PRESTO echo-shifting sequence | 3 | 22.5 | 33.2 | SPM5 | 8 | 40 | . | 4mm isotropic voxel | n/a | n/a |
| Da Silva Alves (da Silva Alves et al. 2011) | Philips Intera | T2*-weighted GE-EPI | 3 | 2000 | 30 | SPM5 | 8 | 35 | 3 | 3x3x3mm | n/a | n/a |
| Nestor (Nestor et al. 2010) | Philips Intera Achieva | T2* weighted EPI | 3 | 2000 | 35 | AFNI | 3mm isotropic rms Gaussian kernel | 32 | 3.5 | . | n/a | n/a |
| Damiano (Damiano et al. 2014) | General Electric Health Technologies 3-T MR750 scanner | SENSE spiral pulse sequence | 3 | 2000 | 30 | Preprocessing- FEAT, MCFLIRT, FLIRT, FMRIB's Improved Linear Model, whole brain FLAME (FMRIB Local Analysis of Mixed Effects) | 5 | 32 | . | 4mm^3^ | Corrected | Clusterwise Z>5.25, ‘corrected’ significance P<0.05 |
| Balodis (Balodis et al. 2012) | Siemens 3 Tesla scanner (Trio; Siemens AG) | T2*-weighted BOLD sequence | 3 | 1500 | 27 | SPM5 | 6 | 25 | 4 | . | Corrected | AlphaSim combined cluster & voxelwise FWE P<0.05 |
| Enzi (Enzi et al. 2012) | 1.5 Siemens Magnetom Symphony (standard imaging head coil) | EPI | 1.5 | 1900 | 45 | SPM5 | 8 | 22 | . | (3×3×3mm) | Corrected | Voxelwise FDR p<0.05 |
| Wu (Wu et al. 2014) | General Electric | T2*-sensitive spiral in/out pulse sequence | 1.5 | 2000 | 40 | AFNI | 4 | 24 | 4 | 3.75x3.75mm (inplane resolution) | Uncorrected | p<0.001, k>16 |
| Kirk (Kirk et al. 2014) | Siemens Trio | EPI | 3 | 2000 | 25 | SPM8 | 8 | 37 | 4 | 3.4x3.4x4.0mm | Corrected | FDR p<0.01, k>10 |
| Romanczuk-Seiferth (Romanczuk-Seiferth et al. 2014) | Siemens Magnetom TIM Trio | GE-EPI | 3 | 2500 | 35 | SPM8 | 8 | 39 | . | 3.5x3.5x3mm | Uncorrected | P<0.001, k>10 |
| Weiland (Weiland et al. 2014) | General Electric Signa | T2*-weighted single shot combined spiral in/out sequences | 3 | 2000 | 30 | SPM8 | 6 | 29 | 4 | . | Corrected | Clusterwise FDR p<0.001 |
| Treadway (Treadway et al. 2013) | Phillips Achieva | GE-EPI | 3 | 2000 | 25 | SPM5 | 6 | 30 | 2.5 | (2mm isotropic voxel) | Corrected | SPM5 cluster correction procedure p<0.05 |
| Vaidya (Vaidya et al. 2013) | Siemens Trio | EPI | 3 | 2000 | 30 | AFNI | 4 | 27 | 3.75 | 3.75x3.75mm inplane resolution  (2mm isotropic voxel) | Corrected | Monte Carlo simulations  using AFNI 3dAlphaSim (uncorrected p<0.005, k>89) corrected alpha of 0.05 |
| Ossewaarde (L. Ossewaarde et al. 2011) | Siemens Avanto | GE-EPI | 1.5 | 2340 | 35 | SPM5 | 8 | 32 | 3.5 | (2x2x2mm) | Corrected | FWE corrected p<0.05 |
| Ossewaarde (Lindsey Ossewaarde et al. 2011) | Siemens TIM Trio | GE-EPI | 3 | 1890 | 25 | SPM5 | 8 | 37 | 3 | (2x2x2mm) | Corrected | FWE corrected p<0.001 |
| Beck (Beck et al. 2009) | Siemens Magnetom Vision | GE-EPI | 1.5 | 1870 | 40 | SPM5 | 8 | 18 | . | 4x4x3.3mm | Uncorrected | P<0.001, k>10 |
| Knutson (Knutson et al. 2003) | General Electric MRI scanner (standard quadrature head coil) | GE | 1.5 | 2000 | 40 | AFNI | 4 | 22 | 3.8 | 3.75x3.75mm inplane resolution | Uncorrected | p<0.0001 |
| De Greck (de Greck et al. 2011) | General Electric Sigma Horizon (standard circular polarized head coil) | GE-EPI | 1.5 | 2000 | 35 | AFNI | . | 23 | 5 | . | Corrected | FDR p<0.0005, k>15 |
| Spreckelmeyer (Spreckelmeyer et al. 2009) | Phillips Medical Systems, Achieva | EPI | 1.5 | 2000 | 50 | SPM5 | 6 | 22 | 3.8 | 4x4x4mm | Corrected | FWE p<0.05 |
| Knutson (Knutson et al. 2008) | General Electric MRI scanner (standard quadrature head coil) | T2*-sensitive in-/out- spiral pulse sequence | 1.5 | . | 40 | AFNI | 4 | 24 | 4 | 4mm^3^ | Uncorrected | P<0.0001 |
| Knutson (Knutson et al. 2001b) | General Electric MRI scanner (standard quadrature head coil) | GE | 1.5 | 2000 | 40 | AFNI | 4 | 16 | 7 | 3.75x3.75mm (inplane resolution) | Uncorrected | P<0.0001 |
| Knutson (Knutson et al. 2001a) | General Electric MRI scanner (standard quadrature head coil) | GE | 1.5 | 2000 | 40 | AFNI | 4 | 16 | 3.8 | 3.75x3.75mm (inplane resolution) | Uncorrected | P<0.05 |

TR (acquisition time), TE (echo time), FWHM (full width at half maximum), FWE (family-wise error corrected), FDR (false discovery rate)

**Supplementary Figure 1: Funnel plots for the highest peaks in AWAN and ALAN with Egger’s test for asymmetry**

a) Anticipation win-vs-neutral: peak 1 for all 33 studies included in omnibus analysis including coordinate-based and group map data with effect size on the x-axis, standard error on the y-axis and Egger’s test beneath
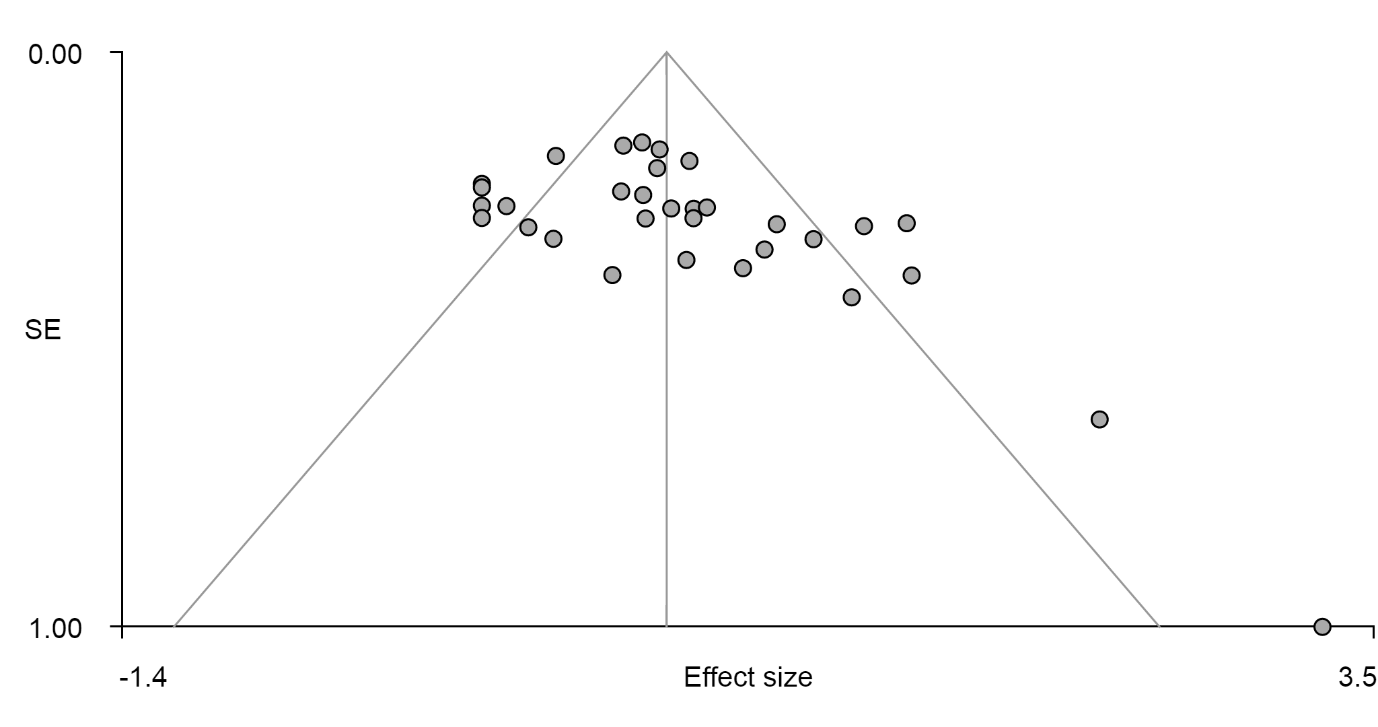


Egger test: Bias: 2.64, t: 2.90, df: 30, **p=0.007**

b) Anticipation win-vs-neutral: peak 1 for all 14 studies involving group map data with effect size on the x-axis, standard error on the y-axis and Egger’s test beneath
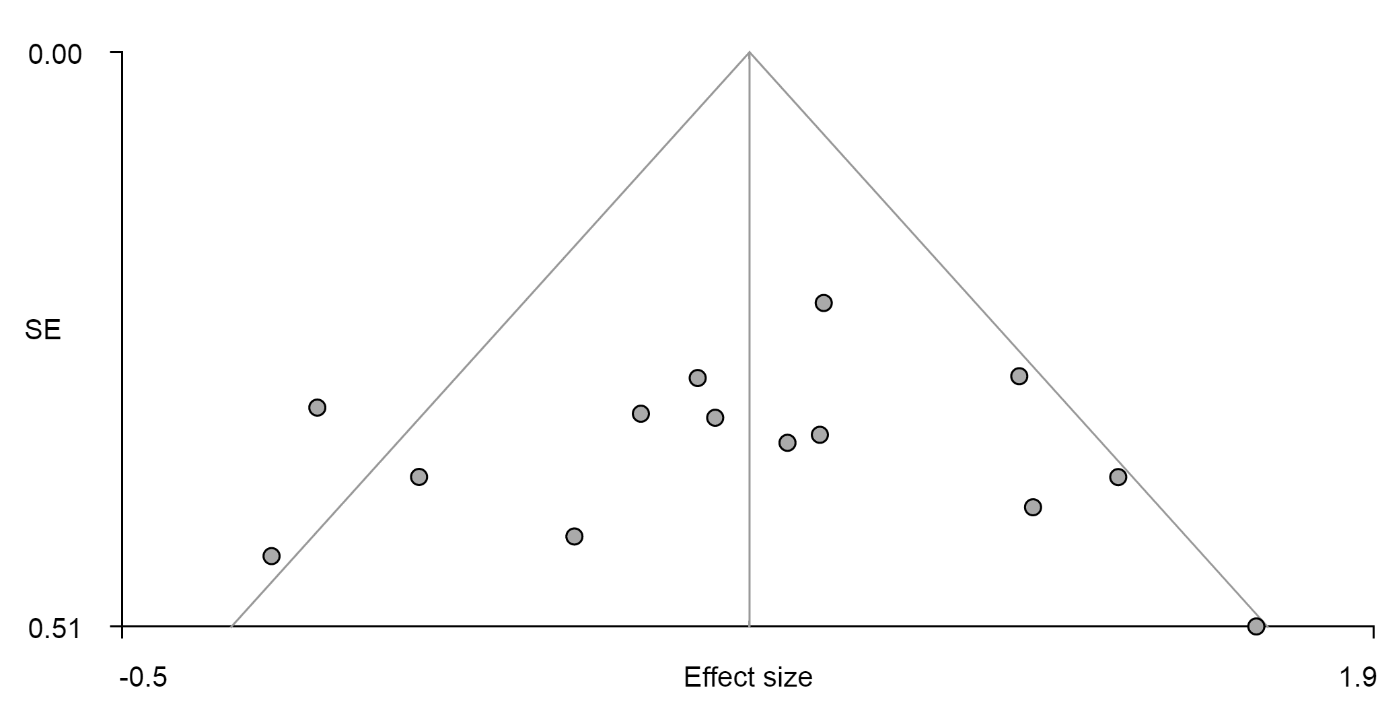


Egger test: Bias: -0.23, t: -0.12, df: 12, p=0.910

c) Anticipation win-vs-neutral: peak 2 for all 33 studies included in omnibus analysis including coordinate-based and group map data with effect size on the x-axis, standard error on the y-axis and Egger’s test beneath


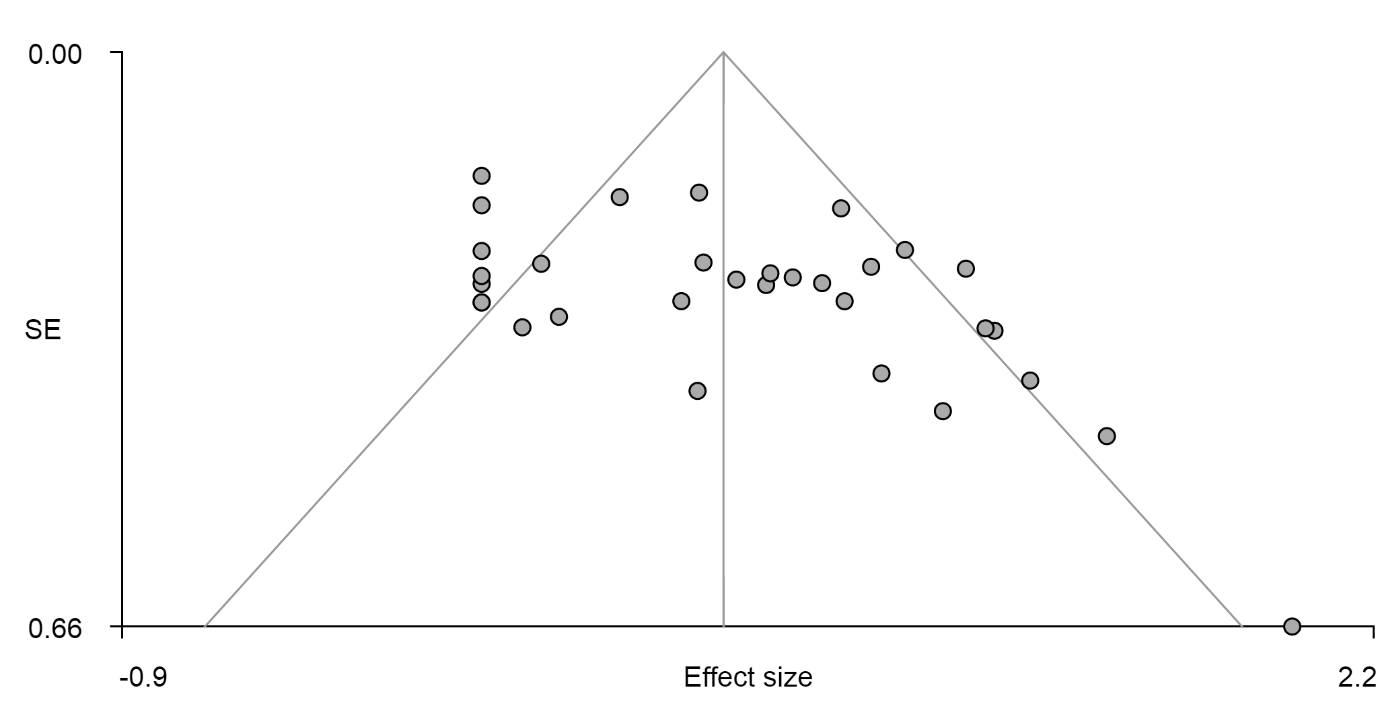


Egger test: Bias: 2.96, t: 2.97, df: 30, **p=0.006**

d) Anticipation win-vs-neutral: peak 2 for all 14 studies involving group map data with effect size on the x-axis, standard error on the y-axis and Egger’s test beneath
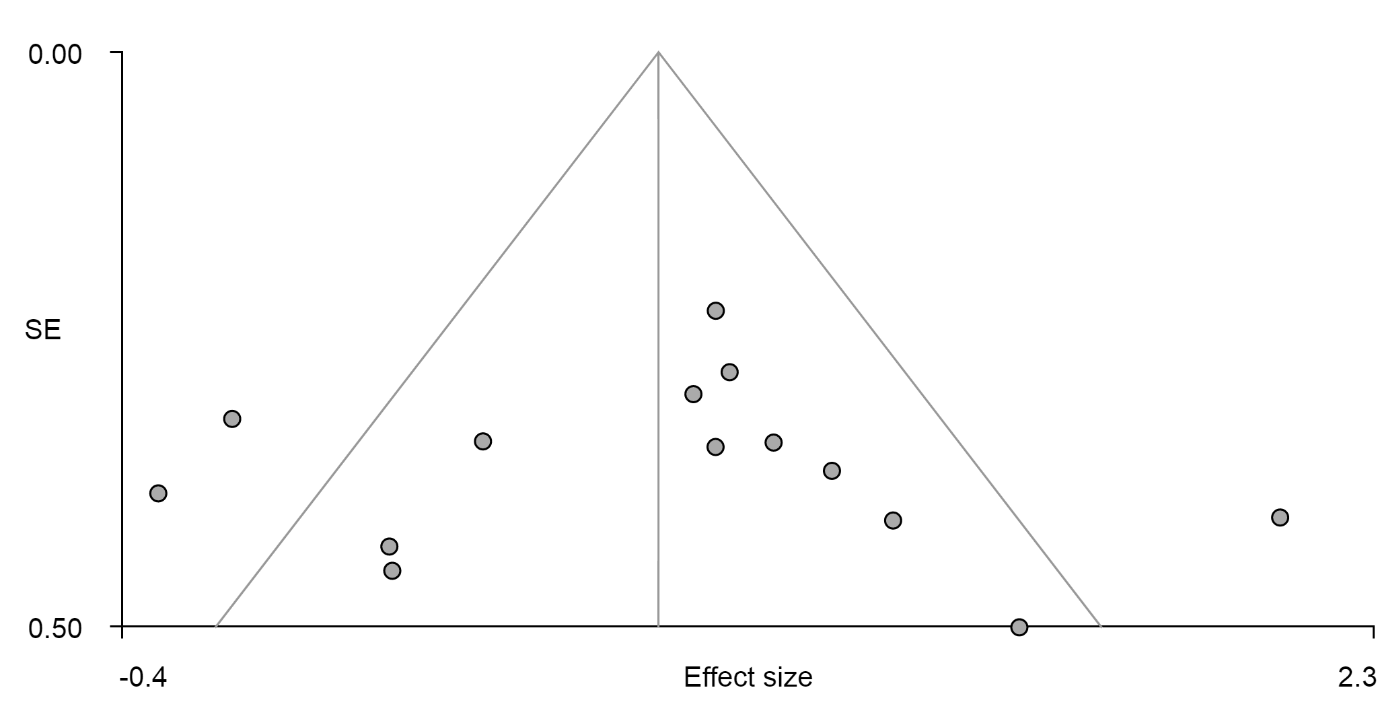


Egger test: Bias: 0.24, t: 0.10, df: 12, p=0.919

e) Anticipation lose-vs-neutral: peak 1 for all 33 studies included in omnibus analysis including coordinate-based and group map data with effect size on the x-axis, standard error on the y-axis and Egger’s test beneath
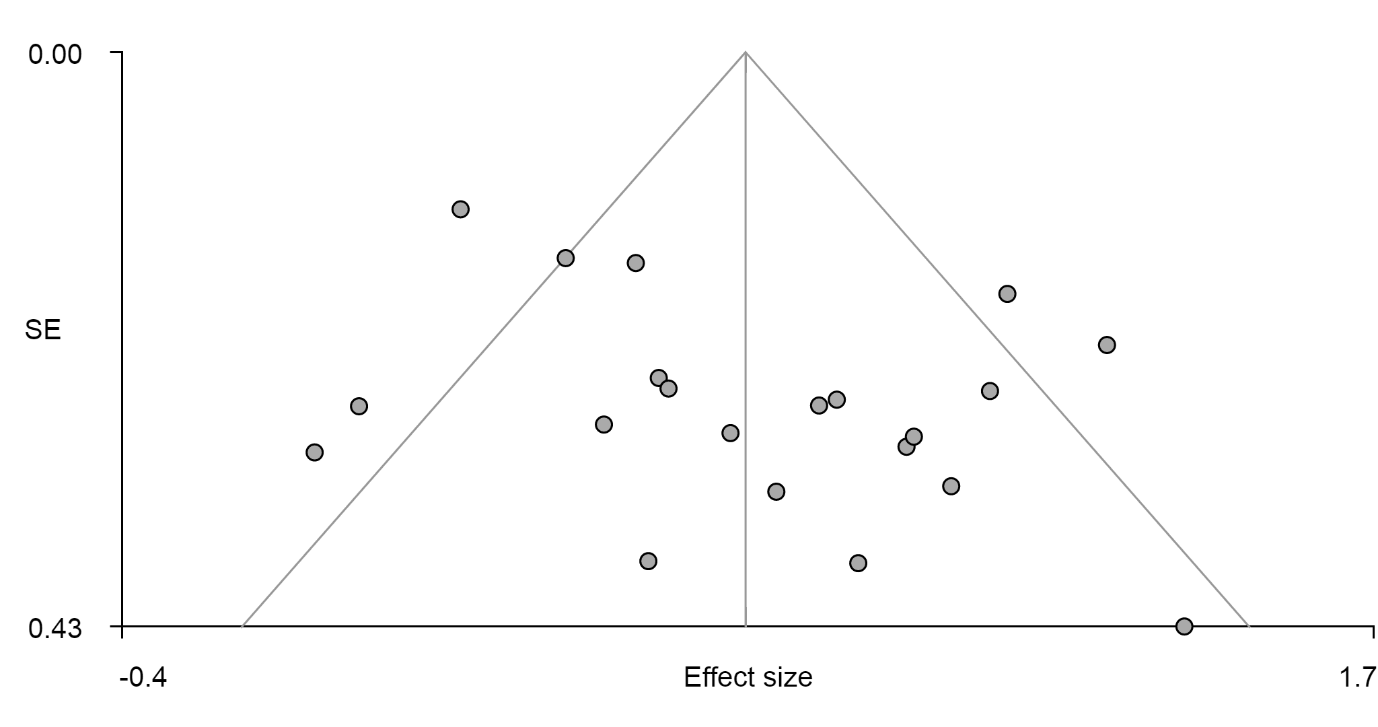


Egger test: Bias: 2.09, t: 2.08, df: 19, **p=0.051**

f) Anticipation lose-vs-neutral: peak 1 for all 11 studies involving group map data with effect size on the x-axis, standard error on the y-axis and Egger’s test beneath
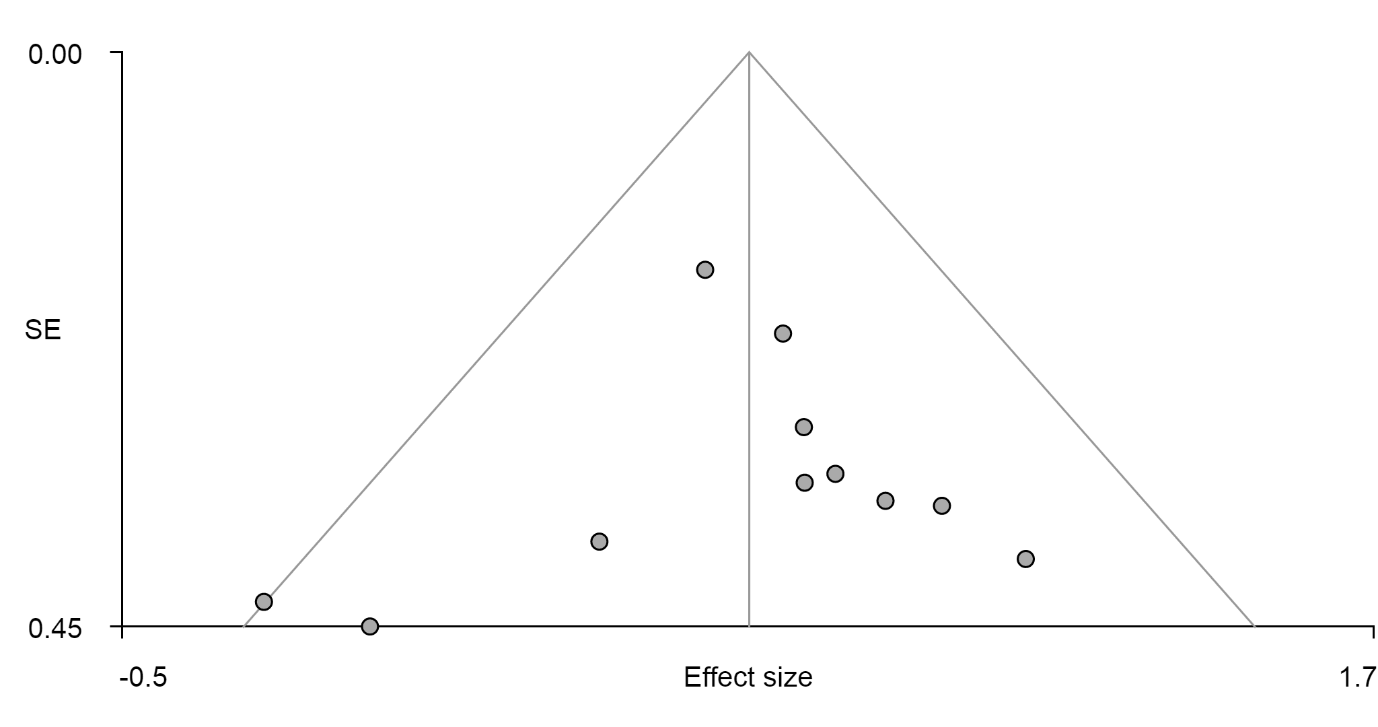


Egger test: Bias: -0.36, t: -0.36, df: 9, p=0.731

**Supplementary Table 4: Omnibus results for text coordinates and maps combined- main peaks**

1. Anticipation win-vs-neutral maps/text activation main peaks

| **Peak MNI coordinate** | **SDM-z** | **P** | **FDR** | **Voxels** | **Anatomical Description** | **Egger’s test p** |
| --- | --- | --- | --- | --- | --- | --- |
| -14,10,0 | 8.552 | ~0 | 0 | 15262 | Left fundus region of putamen | **0.007** |
| 0,8,44 | 7.131 | ~0 | 0 | 5872 | Right anterior cingulate | **0.006** |
| 12,-64,6 | 5.062 | ~0 | 0 | 839 | Right striate area | 0.960 |
| -24,-36,-26 | 4.603 | 0.000196099 | 0.000484 | 401 | Left cerebellum anterior lobe, culmen* | 0.928 |
| -58,-14,22 | 4.505 | 0.000356078 | 0.000738 | 86 | Left postcentral gyrus | 0.723 |
| 52,0,42 | 4.113 | 0.002441049 | 0.003262 | 79 | Right precentral gyrus | 0.124 |
| 26,-6,62 | 4.044 | 0.003349364 | 0.003965 | 26 | Right superior frontal gyrus lateral part | 0.174 |
| 40,54,10 | 4.062 | 0.00312227 | 0.003785 | 21 | Right middle frontal gyrus | 0.691 |
| -20,-48,-34 | 4.082 | 0.002843618 | 0.003625 | 11 | Left cerebellum, anterior lobe* | 0.505 |
| -10,-24,40 | 3.967 | 0.00459826 | 0.004789 | 10 | Left paracentral lobule | 0.066 |
| 44,-6,52 | 3.968 | 0.004567325 | 0.004789 | 10 | Right middle frontal gyrus | 0.313 |

MNI (Montreal Neurological Institute), SDM-z (Signed Differential Mapping z-score), FDR (false discovery rate), *Talairach client

1. Anticipation win-vs-neutral maps/text deactivation main peaks

| **Peak MNI coordinate** | **SDM-z** | **P** | **FDR** | **Voxels** | **Anatomical Description** | **Egger’s test p** |
| --- | --- | --- | --- | --- | --- | --- |
| -52,-64,36 | -3.723 | ~0 | 0 | 5546 | Left angular gyrus | 0.405 |
| -22,24,48 | -2.503 | ~0 | 0 | 4953 | Left superior frontal gyrus, lateral part | 0.917 |
| 56,-62,30 | -3.417 | ~0 | 0 | 1901 | Right superior temporal gyrus | 0.149 |
| 10,-52,30 | -2.521 | ~0 | 0 | 1980 | Right posterior cingulate | 0.486 |
| -48,34,-12 | -2.59 | ~0 | 0 | 1094 | Left inferior frontal gyrus, orbital part | 0.936 |
| 62,-4,-18 | -1.555 | ~0 | 0 | 1542 | Right middle temporal gyrus* | 0.734 |
| 40,-12,14 | -1.249 | 0.000005186 | 0.00000583 | 129 | Right precentral gyrus | 0.381 |
| -28,-14,-24 | -1.001 | 0.000010312 | 0.0000103 | 76 | Left parahippocampal gyrus | 0.770 |
| 0,-38,72 | -1.226 | 0.000005186 | 0.00000583 | 76 | Right postcentral gyrus* | 0.789 |

MNI (Montreal Neurological Institute), SDM-z (Signed Differential Mapping z-score), FDR (false discovery rate), *nearest grey matter structure

1. Anticipation lose-vs-neutral maps/text deactivation all peaks

| **Peak MNI coordinate** | **SDM-z** | **P** | **FDR** | **Voxels** | **Anatomical Description** | **Egger’s test p** |
| --- | --- | --- | --- | --- | --- | --- |
| 16,8,10 | 7.27 | ~0 | 0 | 12127 | Right medial caudate/putamen | **0.051** |
| -6,16,22 | 6.273 | ~0 | 0 | 3500 | Left anterior cingulate gyrus | 0.554 |
| -14,-72,38 | 4.362 | 0.00023222 | 0.000483 | 593 | Left parieto-occipital transition zone | 0.270 |
| -6,-62,4 | 4.085 | 0.000851512 | 0.001352 | 367 | Left occipital gyrus | 0.786 |
| -30,-58,-8 | 4.674 | 0.000067115 | 0.000181 | 204 | Left fusiform gyrus | 0.343 |
| 20,-68,42 | 4.251 | 0.000376761 | 0.000659 | 190 | Right superior parietal lobule | 0.301 |
| 48,-2,42 | 3.841 | 0.002002418 | 0.002313 | 154 | Right precentral gyrus | 0.052 |
| 34,52,4 | 4.267 | 0.000361264 | 0.000655 | 88 | Right middle frontal gyrus | 0.595 |
| 4,-54,-10 | 3.88 | 0.001728892 | 0.002147 | 58 | Right Cerebellum, Anterior Lobe, Culmen* | 0.097 |
| 38,46,20 | 3.842 | 0.001986921 | 0.002313 | 41 | Right middle frontal gyrus | **0.049** |
| 52,12,32 | 4.055 | 0.000985742 | 0.001518 | 38 | Right inferior frontal gyrus, opercular part | 0.441 |
| 22,-44,-8 | 3.93 | 0.001398563 | 0.001906 | 37 | Right fusiform gyrus | 0.854 |
| 30,-82,14 | 3.913 | 0.00151211 | 0.001973 | 30 | Right occipital gyri | 0.161 |
| 10,-78,12 | 3.847 | 0.001940489 | 0.002313 | 29 | Right striate area | 0.158 |
| 24,-80,28 | 3.798 | 0.002353311 | 0.002665 | 28 | Right parieto-occipital transition zone | 0.446 |
| 26,-20,-8 | 4.735 | 0.000046432 | 0.000135 | 17 | Right claustrum | 0.965 |
| -50,-36,38 | 3.675 | 0.003643513 | 0.003712 | 11 | Left supramarginal gyrus | 0.104 |

MNI (Montreal Neurological Institute), SDM-z (Signed Differential Mapping z-score), FDR (false discovery rate), *Talairach client

1. Anticipation lose-vs-neutral maps/text deactivation all peaks

| **Peak MNI coordinate** | **SDM-z** | **P** | **FDR** | **Voxels** | **Anatomical Description** | **Egger’s test p** |
| --- | --- | --- | --- | --- | --- | --- |
| -26,26,48 | -2.168 | ~0 | 0 | 4534 | Left middle frontal gyrus | 0.779 |
| -52,-66,32 | -2.877 | ~0 | 0 | *2334* | Left superior temporal gyrus | 0.174 |
| 58,-62,28 | -2.943 | ~0 | 0 | 2031 | Right superior temporal gyrus | 0.553 |
| -6,-60,28 | -2.178 | ~0 | 0 | 1925 | Left precuneus | 0.194 |
| -60,-12,-16 | -1.663 | 0.000005186 | 0.0000178 | 1081 | Left middle temporal gyrus | 0.410 |
| 60,0,-18 | -1.515 | 0.000020623 | 0.0000527 | 615 | Right middle temporal gyrus | 0.792 |
| -44,38,-18 | -1.776 | 0.000005186 | 0.0000178 | 394 | Left inferior frontal gyrus, orbital part* | 0.739 |
| 2,-56,66 | -1.557 | 0.000010312 | 0.0000334 | 406 | Right precuneus | **0.048** |
| -16,-16,-26 | -1.45 | 0.000025809 | 0.0000619 | 110 | Left entorhinal cortex | 0.097 |
| 34,38,-12 | -1.12 | 0.000185788 | 0.00031 | 60 | Right lateral orbital gyrus | 0.497 |

MNI (Montreal Neurological Institute), SDM-z (Signed Differential Mapping z-score), FDR (false discovery rate), *nearest grey matter structure

**Supplementary Table 5: Only Group Map Results**

1. Anticipation win-vs-neutral activation maps only all peaks

| Peak MNI coordinate | SDM-z | P | FDR | Voxels | Anatomical Description | Egger’s test p |
| --- | --- | --- | --- | --- | --- | --- |
| 2,0,62 | 9.798 | ~0 | 0 | 25114 | Right superior frontal gyrus, lateral part | 0.910 |
| \| MNI coordinate \| SDM-Z \| P \| FDR \| Description \| \| --- \| --- \| --- \| --- \| --- \| \| -2,6,46 \| 9.655 \| ~0 \| 0 \| Left anterior cingulate \| \| -56,2,34 \| 8.871 \| ~0 \| 0 \| Left precentral gyrus \| \| -10,-4,54 \| 8.749 \| ~0 \| 0 \| Left paracentral lobule \| \| 10,6,52 \| 8.711 \| ~0 \| 0 \| Right superior frontal gyrus medial part \| \| -56,2,24 \| 8.652 \| ~0 \| 0 \| Left precentral gyrus \| \| 10,6,12 \| 8.605 \| ~0 \| 0 \| Right medial caudate \| \| 8,14,2 \| 8.554 \| ~0 \| 0 \| Right nucleus accumbens \| \| -12,-2,50 \| 8.511 \| ~0 \| 0 \| Left superior frontal gyrus medial part \| \| 10,12,6 \| 8.417 \| ~0 \| 0 \| Right medial caudate \| \| -10,4,14 \| 8.416 \| ~0 \| 0 \| Left medial caudate \| \| -22,4,8 \| 8.362 \| ~0 \| 0 \| Left putamen \| \| -14,6,12 \| 8.268 \| ~0 \| 0 \| Left medial caudate \| \| -56,0,40 \| 8.261 \| ~0 \| 0 \| Left precentral gyrus \| \| -12,10,34 \| 8.153 \| ~0 \| 0 \| Left anterior cingulate \| \| 26,2,8 \| 8.152 \| ~0 \| 0 \| Right putamen \| \| -22,-2,54 \| 8.145 \| ~0 \| 0 \| Left superior frontal gyrus lateral part \| \| 24,2,0 \| 8.013 \| ~0 \| 0 \| Right putamen \| \| -38,-10,42 \| 7.988 \| ~0 \| 0 \| Left middle frontal gyrus \| \| -4,8,6 \| 7.937 \| ~0 \| 0 \| Left fundus region of caudate \| \| -18,20,2 \| 7.92 \| ~0 \| 0 \| Left putamen \| \| -58,-18,44 \| 7.837 \| ~0 \| 0 \| Left postcentral gyrus \| \| -22,14,0 \| 7.82 \| ~0 \| 0 \| Left putamen \| \| -22,12,-4 \| 7.758 \| ~0 \| 0 \| Left prepiriform claustrum \| \| 2,-28,-2 \| 7.699 \| ~0 \| 0 \| Right periaqueductal grey matter* \| \| -18,6,-2 \| 7.661 \| ~0 \| 0 \| Left putamen \| \| -54,-4,44 \| 7.575 \| ~0 \| 0 \| Left precentral gyrus \| \| -26,-24,58 \| 7.524 \| ~0 \| 0 \| Left postcentral gyrus \| \| -28,-24,64 \| 7.456 \| ~0 \| 0 \| Left postcentral gyrus \| \| 14,-8,14 \| 7.322 \| ~0 \| 0 \| Right medial caudate \| \| -30,24,10 \| 7.274 \| ~0 \| 0 \| Left insular gyrus \| \| -6,4,34 \| 7.252 \| ~0 \| 0 \| Left anterior cingulate \| \| 4,-28,-10 \| 7.223 \| ~0 \| 0 \| Right pretectal area \| \| -18,-66,36 \| 7.217 \| ~0 \| 0 \| Left precuneus \| \| 22,-4,-4 \| 7.167 \| ~0 \| 0 \| Right putamen \| \| 10,12,44 \| 7.136 \| ~0 \| 0 \| Right superior frontal gyrus medial part \| \| -6,16,26 \| 7.121 \| ~0 \| 0 \| Left anterior cingulate \| \| -12,8,0 \| 6.997 \| ~0 \| 0 \| Left fundus region of caudate \| \| -46,-8,54 \| 6.9 \| ~0 \| 0 \| Left precentral gyrus \| \| -16,14,0 \| 6.888 \| ~0 \| 0 \| Left reticular thalamic nucleus* \| \| 38,2,14 \| 6.876 \| ~0 \| 0 \| Right frontal operculum \| \| -32,-44,62 \| 6.864 \| ~0 \| 0 \| Left superior parietal lobule \| \| -52,-10,50 \| 6.838 \| ~0 \| 0 \| Left precentral gyrus \| \| -30,-40,44 \| 6.828 \| ~0 \| 0 \| Left postcentral gyrus \| \| -38,-4,52 \| 6.823 \| ~0 \| 0 \| Left middle frontal gyrus \| \| -54,-14,20 \| 6.82 \| ~0 \| 0 \| Left parietal operculum \| \| 30,-4,48 \| 6.812 \| ~0 \| 0 \| Right middle frontal gyrus \| \| 34,-2,48 \| 6.786 \| ~0 \| 0 \| Right middle frontal gyrus \| \| 38,-2,50 \| 6.773 \| ~0 \| 0 \| Right middle frontal gyrus \| \| -30,-8,66 \| 6.728 \| ~0 \| 0 \| Left superior frontal gyrus lateral part \| \| -14,-78,40 \| 6.706 \| ~0 \| 0 \| Left parieto-occipital transition zone \| \| 14,-22,64 \| 6.642 \| ~0 \| 0 \| Right precentral gyrus \| \| -32,-14,66 \| 6.619 \| ~0 \| 0 \| Left precentral gyrus \| \| 30,-26,48 \| 6.61 \| ~0 \| 0 \| Right precentral gyrus \| \| -42,-28,58 \| 6.593 \| ~0 \| 0 \| Left postcentral gyrus \| \| -38,-32,56 \| 6.591 \| ~0 \| 0 \| Left postcentral gyrus \| \| -26,2,46 \| 6.591 \| ~0 \| 0 \| Left middle frontal gyrus \| \| 36,-20,40 \| 6.584 \| ~0 \| 0 \| Right postcentral gyrus \| \| -10,-16,14 \| 6.574 \| ~0 \| 0 \| Left ventral lateral posterior thalamic \| \| -34,-34,62 \| 6.567 \| ~0 \| 0 \| Left postcentral gyrus \| \| 46,-4,52 \| 6.562 \| ~0 \| 0 \| Right middle frontal gyrus \| \| 24,-70,6 \| 6.552 \| ~0 \| 0 \| Right occiptal gyri \| \| -26,-32,54 \| 6.534 \| ~0 \| 0 \| Left postcentral gyrus \| \| -8,-26,12 \| 6.517 \| ~0 \| 0 \| Left medial dorsal thalamic nucleus \| \| -18,-74,40 \| 6.505 \| ~0 \| 0 \| Left parieto-occipital transition zone \| \| -12,-30,60 \| 6.498 \| ~0 \| 0 \| Left precentral gyrus \| \| -2,0,12 \| 6.486 \| ~0 \| 0 \| Left medial septal nucleus \| \| -14,-24,12 \| 6.471 \| ~0 \| 0 \| Left pulvinar \| \| -48,-24,48 \| 6.447 \| ~0 \| 0 \| Left postcentral gyrus \| \| 6,-30,-18 \| 6.435 \| ~0 \| 0 \| Right cerebellum anterior lobe culmen** \| \| -54,-22,44 \| 6.352 \| ~0 \| 0 \| Left posterior central gyrus \| \| 14,-14,40 \| 6.332 \| ~0 \| 0 \| Right posterior cingulate \| \| -10,-12,8 \| 6.275 \| ~0 \| 0 \| Left ventral anterior thalamic nucleus \| \| 20,18,-8 \| 6.187 \| ~0 \| 0 \| Right posterior orbital gyrus \| \| -12,-34,66 \| 6.181 \| ~0 \| 0 \| Left postcentral gyrus \| \| 2,-16,-2 \| 6.179 \| ~0 \| 0 \| Right ventral anterior thalamic nucleus** \| \| 10,-40,-14 \| 6.152 \| ~0 \| 0 \| Right cerebellum anterior lobe culmen** \| \| -2,-18,52 \| 6.133 \| ~0 \| 0 \| Left paracentral lobule \| \| -24,4,66 \| 6.114 \| ~0 \| 0 \| Left superior frontal gyrus lateral part \| \| 24,-74,22 \| 6.098 \| ~0 \| 0 \| Right precuneus \| \| -42,0,10 \| 6.09 \| ~0 \| 0 \| Left frontal operculum \| \| 8,34,30 \| 6.081 \| ~0 \| 0 \| Right anterior cingulate \| \| -48,-20,56 \| 6.067 \| ~0 \| 0 \| Left postcentral gyrus \| \| -2,-30,-20 \| 6.06 \| ~0 \| 0 \| Left cerebellum anterior lobe culmen** \| \| 14,-32,60 \| 6.047 \| ~0 \| 0 \| Right postcentral gyrus \| \| -46,-34,40 \| 6.039 \| ~0 \| 0 \| Left supramarginal gyrus \| \| -26,-76,28 \| 6.023 \| ~0 \| 0 \| Left parieto-occipital transition zone \| \| -6,-34,-14 \| 6.009 \| ~0 \| 0 \| Left cerebellum anterior lobe culmen** \| \| 44,-6,58 \| 5.991 \| ~0 \| 0 \| Right middle frontal gyrus \| \| 12,-30,56 \| 5.963 \| ~0 \| 0 \| Right precentral gyrus \| \| 14,-32,64 \| 5.952 \| ~0 \| 0 \| Right postcentral gyrus \| \| 26,-6,58 \| 5.948 \| ~0 \| 0 \| Right superior frontal gyrus \| \| 30,-54,38 \| 5.948 \| ~0 \| 0 \| Right superior parietal lobule \| \| -12,-20,0 \| 5.921 \| ~0 \| 0 \| Left paracentral lobule \| \| 14,-44,-18 \| 5.905 \| ~0 \| 0 \| Right cerebellum anterior lobe culmen** \| \| -8,-32,54 \| 5.889 \| ~0 \| 0 \| Left precenral gyrus \| \| -48,-36,52 \| 5.865 \| ~0 \| 0 \| Left supramarginal gyrus \| \| -10,-36,52 \| 5.85 \| ~0 \| 0 \| Left paracentral lobule \| \| -40,-34,42 \| 5.824 \| ~0 \| 0 \| Left supramarginal gyrus \| \| -28,-6,4 \| 5.809 \| ~0 \| 0 \| Left putamen \| \| 8,-20,10 \| 5.807 \| ~0 \| 0 \| Right medial dorsal thalamic \| \| 38,-66,16 \| 5.738 \| ~0 \| 0 \| Right middle temporal gyrus \| \| -4,-26,64 \| 5.711 \| ~0 \| 0 \| Left paracentral lobule \| \| -34,4,4 \| 5.709 \| ~0 \| 0 \| Left claustrum \| \| 6,14,26 \| 5.699 \| ~0 \| 0 \| Right anterior cingulate \| \| -16,-60,52 \| 5.62 \| ~0 \| 0 \| Left superior parietal lobule \| \| -60,2,12 \| 5.615 \| ~0 \| 0 \| Left precentral gyrus \| \| -18,-14,22 \| 5.594 \| ~0 \| 0 \| Left caudate \| \| 28,-54,52 \| 5.57 \| ~0 \| 0 \| Right superior parietal lobule \| \| 30,-76,26 \| 5.566 \| ~0 \| 0 \| Right angular gyrus \| \| -24,-56,48 \| 5.543 \| ~0 \| 0 \| Left superior parietal lobule \| \| 48,-18,40 \| 5.473 \| ~0 \| 0 \| Right precentral gyrus \| \| -52,2,0 \| 5.458 \| ~0 \| 0 \| Left planum polare \| \| -18,-10,20 \| 5.443 \| ~0 \| 0 \| Left caudate \| \| 54,12,32 \| 5.423 \| ~0 \| 0 \| Right inferior frontal gyrus opercular part \| \| -18,-16,70 \| 5.421 \| ~0 \| 0 \| Left precentral gyrus \| \| -26,-54,42 \| 5.42 \| ~0 \| 0 \| Left superior parietal lobule \| \| -12,-30,72 \| 5.389 \| ~0 \| 0 \| Left postcentral gyrus \| \| 36,26,8 \| 5.347 \| ~0 \| 0 \| Right inferior frontal gyrus orbital part \| \| 12,-42,48 \| 5.321 \| ~0 \| 0 \| Right paracentral lobule \| \| 56,8,38 \| 5.312 \| ~0 \| 0 \| Right inferior frontal gyrus opercular part \| \| 8,22,26 \| 5.299 \| ~0 \| 0 \| Right subcallosal gyrus \| \| 30,24,6 \| 5.295 \| ~0 \| 0 \| Right insular gyrus \| \| -52,-22,20 \| 5.287 \| ~0 \| 0 \| Left parietal operculum \| \| 8,-48,-10 \| 5.243 \| ~0 \| 0 \| Right cerebellum anterior lobe culmen** \| \| 0,-48,-18 \| 5.229 \| ~0 \| 0 \| Right posterior cingulate \| \| 34,-74,14 \| 5.218 \| ~0 \| 0 \| Right middle temporal gyrus \| \| 36,22,12 \| 5.203 \| ~0 \| 0 \| Right inferior frontal gyrus triangular part \| \| 28,-50,44 \| 5.196 \| ~0 \| 0 \| Right superior parietal lobule \| \| 58,8,14 \| 5.178 \| ~0 \| 0 \| Right precentral gyrus \| \| 22,-64,42 \| 5.148 \| ~0 \| 0 \| Right superior parietal lobule \| \| 14,-30,46 \| 5.118 \| ~0 \| 0 \| Right paracentral lobule \| \| 32,-76,10 \| 5.094 \| ~0 \| 0 \| Right occiptal gyri* \| \| 22,-64,46 \| 5.088 \| ~0 \| 0 \| Right superior parietal lobule \| \| 36,-74,10 \| 5.088 \| ~0 \| 0 \| Right middle temporal gyrus \| \| 4,-54,-10 \| 5.087 \| ~0 \| 0 \| Right cerebellum anterior lobe culmen** \| \| 16,-64,52 \| 5.079 \| ~0 \| 0 \| Right superior parietal lobule \| \| 38,-28,46 \| 5.073 \| ~0 \| 0 \| Right postcentral gyrus \| \| -26,-66,52 \| 5.059 \| ~0 \| 0 \| Left superior parietal lobule \| \| 26,-62,34 \| 5.034 \| ~0 \| 0 \| Right angular gyrus \| \| 18,-66,34 \| 5.014 \| ~0 \| 0 \| Right precuneus \| \| -8,36,16 \| 4.986 \| 0.0011096 \| 0.001370382 \|  \| \| -10,-10,-14 \| 4.975 \| 0.001140535 \| 0.001402126 \|  \| \| 38,-70,20 \| 4.957 \| 0.001186967 \| 0.001439399 \|  \| \| 18,-26,8 \| 4.951 \| 0.001186967 \| 0.001439399 \|  \| \| 18,-10,70 \| 4.946 \| 0.001217961 \| 0.00146301 \|  \| \| 22,-62,32 \| 4.936 \| 0.001228273 \| 0.00146301 \|  \| \| -10,-70,46 \| 4.93 \| 0.001238585 \| 0.001468765 \|  \| \| 14,-32,40 \| 4.913 \| 0.001290202 \| 0.001523234 \|  \| \| 18,-60,4 \| 4.911 \| 0.001305699 \| 0.001534769 \|  \| \| 12,-64,54 \| 4.907 \| 0.001321197 \| 0.001546204 \|  \| \| 52,8,2 \| 4.841 \| 0.001491487 \| 0.001730383 \|  \| \| 12,-12,50 \| 4.825 \| 0.001579225 \| 0.001808685 \|  \| \| 18,-28,4 \| 4.823 \| 0.001605034 \| 0.001830422 \|  \| \| 52,0,16 \| 4.814 \| 0.001641154 \| 0.001863683 \|  \| \| 0,-22,-18 \| 4.789 \| 0.00174433 \| 0.001964204 \|  \| \| -18,-88,18 \| 4.774 \| 0.001806259 \| 0.00202278 \|  \| \| 12,-78,12 \| 4.733 \| 0.001961112 \| 0.002180822 \|  \| \| 42,6,22 \| 4.711 \| 0.002038538 \| 0.002257554 \|  \| \| 14,-72,36 \| 4.666 \| 0.002275944 \| 0.002499807 \|  \| \| 40,10,22 \| 4.657 \| 0.002301693 \| 0.00251777 \|  \| \| 24,-60,28 \| 4.649 \| 0.002342999 \| 0.002542201 \|  \| \| 16,-58,48 \| 4.643 \| 0.002379119 \| 0.002570983 \|  \| \| 10,-74,38 \| 4.628 \| 0.002482355 \| 0.002671772 \|  \| \| 46,4,18 \| 4.608 \| 0.00258559 \| 0.002744316 \|  \| \| 18,-50,56 \| 4.582 \| 0.002750695 \| 0.002857311 \|  \| \| 6,-6,32 \| 4.51 \| 0.003210008 \| 0.003308777 \|  \| \| 16,-76,40 \| 4.479 \| 0.003478408 \| 0.003517786 \|  \| \| 48,6,26 \| 4.457 \| 0.003653884 \| 0.003681357 \|  \| \| 34,-46,56 \| 4.387 \| 0.004293799 \| 0.004309881 \|  \| | | | | | | |
| 34,40,26 | 6.424 | ~0 | 0 | 405 | Right middle frontal gyrus | 0.919 |
| \| 40,54,6 \| 5.306 \| ~0 \| 0 \| Right middle frontal gyrus \| \| --- \| --- \| --- \| --- \| --- \| \| 34,40,34 \| 5.123 \| ~0 \| 0 \| Right middle frontal gyrus \| \| 32,54,12 \| 4.945 \| 0.001223087 \| 0.00146301 \|  \| \| -34,42,28 \| 6.679 \| ~0 \| 0 \|  \| \| -32,38,20 \| 5.437 \| ~0 \| 0 \|  \| \| -34,52,14 \| 4.829 \| 0.001553416 \| 0.001786762 \|  \| \| -32,60,0 \| 4.79 \| 0.001739204 \| 0.001964204 \|  \| \| -34,56,0 \| 4.688 \| 0.002172709 \| 0.002396239 \|  \| \| -32,44,10 \| 4.48 \| 0.003452599 \| 0.003504911 \|  \| | | | | | | |
| -34,42,28 | 6.679 | ~0 | 0 | 313 | Left middle frontal gyrus | 0.248 |
| \| -32,38,20 \| 5.437 \| ~0 \| 0 \| Left middle frontopolar gyrus \| \| --- \| --- \| --- \| --- \| --- \| \| -34,52,14 \| 4.829 \| 0.001553416 \| 0.001786762 \|  \| \| -32,60,0 \| 4.79 \| 0.001739204 \| 0.001964204 \|  \| \| -34,56,0 \| 4.688 \| 0.002172709 \| 0.002396239 \|  \| \| -32,44,10 \| 4.48 \| 0.003452599 \| 0.003504911 \|  \| | | | | | | |
| -16,-22,38 | 6.322 | ~0 | 0 | 124 | Left paracentral lobule | 0.097 |
| 46,-52,-10 | 5.699 | ~0 | 0 | 125 | Right inferior temporal gyrus | 0.139 |
| \| 40,-56,-8 \| 5.495 \| ~0 \| 0 \| Right inferior temporal gyrus \| \| --- \| --- \| --- \| --- \| --- \| \| 46,-46,-10 \| 4.956 \| 0.001186967 \| 0.001439399 \|  \| | | | | | | |
| -20,-72,10 | 5.476 | ~0 | 0 | 83 | Left occipital gyri | 0.123 |
| \| -20,-70,4 \| 4.604 \| 0.002590716 \| 0.002744316 \| \| --- \| --- \| --- \| --- \| | | | | | | |
| -40,-56,-6 | 5.440 | ~0 | 0 | 51 | Left inferior temporal gyrus | 0.354 |
| -18,-42,-6 | 5.437 | ~0 | 0 | 26 | Left parahippocampal gyrus | 0.910 |
| -6,-30,28 | 4.082 | ~0 | 0 | 19 | Left posterior cingulate | 0.184 |
| -24,-56,8 | 3.967 | ~0 | 0 | 18 | Left striate | 0.864 |
| -44,-42,-12 | 3.968 | ~0 | 0 | 10 | Left planum polare | 0.740 |

MNI (Montreal Neurological Institute), SDM-z (Signed Differential Mapping z-score), FDR (false discovery rate), *nearest grey matter structure, **Talairach client

1. Anticipation win-vs-neutral maps only deactivation all peaks

| **Peak MNI coordinate** | **SDM-z** | **P** | **FDR** | **Voxels** | **Anatomical Description** | **Egger’s test p** |
| --- | --- | --- | --- | --- | --- | --- |
| -52,-64,36 | -6.434 | ~0 | 0 | 4857 | Left angular gyrus | 0.670 |
| \| -58,-58,28 \| -4.852 \| ~0 \| 0 \| \| --- \| --- \| --- \| --- \| \| -58,-62,22 \| -4.649 \| ~0 \| 0 \| \| -60,-18,-12 \| -3.097 \| ~0 \| 0 \| \| -56,0,-24 \| -2.751 \| ~0 \| 0 \| \| -58,-10,-14 \| -2.724 \| ~0 \| 0 \| \| -62,-10,-20 \| -2.709 \| ~0 \| 0 \| \| -60,-32,-8 \| -2.506 \| 0.000005186 \| 7.64E-06 \| \| -54,6,-30 \| -2.484 \| 0.000005186 \| 7.64E-06 \| \| -60,-26,-10 \| -2.306 \| 0.000005186 \| 7.64E-06 \| \| -68,-50,4 \| -1.105 \| 0.000356078 \| 0.000465507 \| \| -60,-50,44 \| -0.7 \| 0.001537919 \| 0.001776562 \| \| -60,-68,6 \| -0.461 \| 0.003297746 \| 0.003386191 \| | | | | | | |
| -44,36,-12 | -4.623 | ~0 | 0 | 5739 | Left inferior frontal gyrus orbital part | 0.074 |
| \| -40,16,48 \| -4.104 \| ~0 \| 0 \| \| --- \| --- \| --- \| --- \| \| -22,24,48 \| -3.667 \| ~0 \| 0 \| \| -56,24,14 \| -3.035 \| ~0 \| 0 \| \| -16,48,34 \| -3.031 \| ~0 \| 0 \| \| -12,30,54 \| -2.617 \| ~0 \| 0 \| \| -14,60,18 \| -2.424 \| 0.000005186 \| 7.64E-06 \| \| 20,26,48 \| -2.319 \| 0.000005186 \| 7.64E-06 \| \| 20,28,52 \| -2.262 \| 0.000005186 \| 7.64E-06 \| \| -4,58,20 \| -2.231 \| 0.000010312 \| 1.51E-05 \| \| -12,36,48 \| -1.922 \| 0.000025809 \| 3.72E-05 \| \| -16,54,26 \| -1.789 \| 0.000041306 \| 5.89E-05 \| \| -4,56,-8 \| -1.538 \| 0.000092924 \| 0.000131072 \| \| 18,42,46 \| -1.513 \| 0.00009805 \| 0.000136152 \| \| 16,46,42 \| -1.357 \| 0.000165164 \| 0.000225837 \| \| 36,24,48 \| -1.277 \| 0.000221908 \| 0.000298851 \| \| 12,54,34 \| -1.256 \| 0.000237405 \| 0.00031654 \| \| 10,56,26 \| -1.034 \| 0.000474811 \| 0.000611776 \| \| -52,22,32 \| -0.647 \| 0.001811445 \| 0.00202278 \| \| 44,16,50 \| -0.336 \| 0.004613757 \| 0.004613757 \| | | | | | | |
| 54,-62,30 | -5.253 | ~0 | 0 | 1736 | Right superior temporal gyrus | 0.613 |
| \| 58,-58,30 \| -5.248 \| ~0 \| 0 \| Right superior temporal gyrus \| \| --- \| --- \| --- \| --- \| --- \| \| 42,-54,22 \| -2.989 \| ~0 \| 0 \|  \| | | | | | | |
| -8,-52,32 | -3.102 | ~0 | 0 | 2159 | Left precuneus | 0.756 |
| \| 8,-52,32 \| -3.068 \| ~0 \| 0 \| \| --- \| --- \| --- \| --- \| \| 0,-60,30 \| -2.934 \| ~0 \| 0 \| \| 12,-52,12 \| -1.985 \| 0.000025809 \| 3.72E-05 \| \| -10,-54,14 \| -1.923 \| 0.000025809 \| 3.72E-05 \| | | | | | | |
| 62,-10,-20 | -1.797 | 0.000041306 | 5.89E-05 | 807 | Right middle temporal gyrus | 0.570 |
| \| 60,-4,-16 \| -1.77 \| 0.000046432 \| 6.58E-05 \| \| --- \| --- \| --- \| --- \| \| 54,-8,-28 \| -1.525 \| 0.00009805 \| 0.000136152 \| \| 48,6,-42 \| -1.497 \| 0.00009805 \| 0.000136152 \| \| 50,4,-28 \| -1.243 \| 0.000247717 \| 0.000327035 \| \| 48,0,-32 \| -1.088 \| 0.000376761 \| 0.000490155 \| \| 46,-12,-32 \| -0.873 \| 0.000846386 \| 0.00105503 \| \| 52,-8,-34 \| -0.867 \| 0.000846386 \| 0.00105503 \| \| 48,-16,-36 \| -0.757 \| 0.001217961 \| 0.00146301 \| \| 48,-8,-40 \| -0.548 \| 0.002518475 \| 0.002689049 \| \| 66,-18,-20 \| -0.443 \| 0.003442287 \| 0.003504911 \| | | | | | | |
| 40,-12,12 | -2.812 | ~0 | 0 | 228 | Right precentral gyrus | 0.928 |
| 26,-18,-24 | -1.421 | 0.000139356 | 0.000192512 | 353 | Right parahippocampal gyrus | 0.750 |
| \| 28,-10,-24 \| -1.347 \| 0.00017029 \| 0.000230494 \| \| --- \| --- \| --- \| --- \| \| 22,-6,-24 \| -1.261 \| 0.000227094 \| 0.000304306 \| | | | | | | |
| -24,-8,-24 | -2.488 | 0.000005186 | 7.64E-06 | 213 | Left parahippocampal gyrus | 0.842 |
| 18,-84,-48 | -1.022 | 0.000521243 | 0.000662053 | 236 | Right cerebellum posterior lobe inferior semi-lunar lobule** | 0.297 |
| \| 28,-82,-50 \| -1.007 \| 0.000552177 \| 0.000698035 \| \| --- \| --- \| --- \| --- \| \| 22,-84,-48 \| -0.981 \| 0.000583172 \| 0.000733756 \| \| 40,-70,-54 \| -0.573 \| 0.002327502 \| 0.002535653 \| \| 42,-66,-48 \| -0.526 \| 0.002662957 \| 0.002787783 \| \| 42,-62,-48 \| -0.456 \| 0.003344178 \| 0.003420762 \| | | | | | | |
| 0,32,0 | -1.185 | 0.000289023 | 0.000379697 | 90 | Left anterior cingulate | 0.608 |
| \| 0,24,-8 \| -0.506 \| 0.002859116 \| 0.002958468 \| \| --- \| --- \| --- \| --- \| | | | | | | |
| 0,-38,72 | -1.35 | 0.00017029 | 0.000230494 | 63 | Left paracentral lobule | 0.252 |
| \| 2,-48,70 \| -0.531 \| 0.002642334 \| 0.002787783 \| \| --- \| --- \| --- \| --- \| \| 6,-50,70 \| -0.527 \| 0.002662957 \| 0.002787783 \| \| 2,-60,64 \| -0.517 \| 0.002750695 \| 0.002857311 \| | | | | | | |
| -24,-40,8 | -1.401 | 0.000149667 | 0.000205696 | 42 | Left dentate gyrus | 0.116 |
| \| -24,-42,4 \| -1.249 \| 0.000242531 \| 0.000321774 \| \| --- \| --- \| --- \| --- \| \| -20,-38,12 \| -1.056 \| 0.00043869 \| 0.000567966 \| \| -32,-46,0 \| -1.027 \| 0.000495434 \| 0.000635293 \| \| -16,-36,12 \| -0.807 \| 0.001057982 \| 0.001312681 \| | | | | | | |
| 32,6,-36 | -1.022 | 0.000521243 | 0.000665625 | 36 | Right fusiform gyrus* | **0.039** |

MNI (Montreal Neurological Institute), SDM-z (Signed Differential Mapping z-score), FDR (false discovery rate), *nearest grey matter structure, **Talairach client

1. Anticipation lose-vs-neutral activation maps only all peaks

| Peak MNI coordinate | | SDM-z | | P | | FDR | | | | | Voxels | Anatomical Description | | | | | | Egger’s test | |
| --- | --- | --- | --- | --- | --- | --- | --- | --- | --- | --- | --- | --- | --- | --- | --- | --- | --- | --- | --- |
| 0,18,52 | | 6.801 | | ~0 | | 0 | | | | | 11315 | Left superior frontal gyrus medial part | | | | | | 0.731 | |
| \| 2,-12,-2 \| 6.254 \| ~0 \| 0 \| Right posterior hypothalamic area \| \| --- \| --- \| --- \| --- \| --- \| \| 32,-20,0 \| 6.019 \| ~0 \| 0 \| Right putamen \| \| -24,2,6 \| 5.993 \| ~0 \| 0 \| Left putamen \| \| 6,-12,30 \| 5.978 \| ~0 \| 0 \| Right posterior cingulate \| \| -58,2,28 \| 5.801 \| ~0 \| 0 \| Left precentral gyrus \| \| 2,-28,-2 \| 5.793 \| ~0 \| 0 \| Right pretectal area \| \| -46,4,20 \| 5.793 \| ~0 \| 0 \| Left inferior frontal gyrus opercular part \| \| -52,6,44 \| 5.75 \| ~0 \| 0 \| Left precentral gyrus \| \| -48,12,18 \| 5.739 \| ~0 \| 0 \| Left inferior frontal gyrus opercular part \| \| -26,8,-2 \| 5.672 \| ~0 \| 0 \| Left prepiriform claustrum \| \| -14,-22,-2 \| 5.666 \| ~0 \| 0 \| Left ventroposterior inferior thalamic nucleus \| \| -6,2,30 \| 5.657 \| ~0 \| 0 \| Left anterior cingulate \| \| 12,-12,12 \| 5.634 \| ~0 \| 0 \| Right ventroanterior thalamic nucleus \| \| 28,-6,8 \| 5.61 \| ~0 \| 0 \| Right putamen \| \| -22,8,-2 \| 5.593 \| ~0 \| 0 \| Left putamen \| \| 26,-2,8 \| 5.567 \| ~0 \| 0 \| Right putamen \| \| 32,14,8 \| 5.554 \| ~0 \| 0 \| Right insula \| \| -12,-20,2 \| 5.518 \| ~0 \| 0 \| Left ventrolateral posterior thalamic nucleus \| \| 16,16,2 \| 5.48 \| ~0 \| 0 \| Right putamen \| \| 40,30,2 \| 5.474 \| ~0 \| 0 \| Right inferior frontal gyrus triangular part \| \| 52,12,34 \| 5.444 \| ~0 \| 0 \| Right inferior frontal gyrus opercular part \| \| -14,-18,12 \| 5.429 \| ~0 \| 0 \| Left ventrolateral posterior thalamic nucleus \| \| 20,2,6 \| 5.35 \| ~0 \| 0 \| Right putamen \| \| 26,2,8 \| 5.338 \| ~0 \| 0 \| Right putamen \| \| 16,8,10 \| 5.337 \| ~0 \| 0 \| Right caudate \| \| 6,12,56 \| 5.327 \| ~0 \| 0 \| Right superior frontal gyrus medial part \| \| 30,0,12 \| 5.325 \| ~0 \| 0 \| Right claustrum \| \| -18,-28,8 \| 5.187 \| ~0 \| 0 \| Left lateral pulvinar \| \| 2,28,48 \| 5.181 \| ~0 \| 0 \| Left superior frontal gyrus medial part \| \| 12,-30,56 \| 5.172 \| ~0 \| 0 \| Right precentral gyrus \| \| -28,-10,60 \| 5.167 \| ~0 \| 0 \| Left superior frontal gyrus lateral part \| \| -4,8,56 \| 5.161 \| ~0 \| 0 \| Left superior frontal gyrus medial part \| \| -8,6,46 \| 5.159 \| ~0 \| 0 \| Left superior frontal gyrus medial part \| \| -4,2,62 \| 5.157 \| ~0 \| 0 \| Left superior frontal gyrus medial part \| \| 38,30,8 \| 5.126 \| ~0 \| 0 \| Right inferior frontal gyrus triangular part \| \| -16,-6,-12 \| 5.12 \| ~0 \| 0 \| Left amygdala (basomedial nucleus) \| \| -18,-24,10 \| 5.094 \| ~0 \| 0 \| Left ventroposterior lateral thalamic nucleus \| \| -6,18,22 \| 5.082 \| ~0 \| 0 \| Left anterior cingulate \| \| -4,6,62 \| 5.08 \| ~0 \| 0 \| Left superior frontal gyrus medial part \| \| -4,10,26 \| 5.067 \| ~0 \| 0 \| Left anterior cingulate \| \| -12,12,42 \| 5.056 \| ~0 \| 0 \| Left superior frontal gyrus medial part \| \| -50,0,14 \| 5.052 \| ~0 \| 0 \| Left precentral gyrus \| \| -4,6,44 \| 5.05 \| ~0 \| 0 \| Left anterior cingulate \| \| -22,-22,58 \| 5.037 \| ~0 \| 0 \| Left postcentral gyrus \| \| 20,-26,10 \| 5.036 \| ~0 \| 0 \| Right ventroposterior lateral thalamic nucleus \| \| 8,-2,64 \| 5.026 \| ~0 \| 0 \| Right superior frontal gyrus lateral part \| \| -46,-2,48 \| 5.014 \| ~0 \| 0 \| Left middle frontal gyrus \| \| 40,12,24 \| 4.993 \| 0.000154853 \| 0.000343742 \|  \| \| -10,14,0 \| 4.986 \| 0.000154853 \| 0.000343742 \|  \| \| 30,-12,8 \| 4.979 \| 0.000159979 \| 0.000343742 \|  \| \| -4,-24,64 \| 4.975 \| 0.000159979 \| 0.000343742 \|  \| \| -52,-36,36 \| 4.968 \| 0.000159979 \| 0.000343742 \|  \| \| -12,8,12 \| 4.968 \| 0.000159979 \| 0.000343742 \|  \| \| 2,0,64 \| 4.918 \| 0.00017029 \| 0.000358902 \|  \| \| 26,18,0 \| 4.916 \| 0.000175476 \| 0.000360701 \|  \| \| 28,4,54 \| 4.896 \| 0.000180602 \| 0.00036309 \|  \| \| 44,16,8 \| 4.889 \| 0.000180602 \| 0.00036309 \|  \| \| -14,10,0 \| 4.886 \| 0.000180602 \| 0.00036309 \|  \| \| 12,-16,38 \| 4.868 \| 0.000190973 \| 0.000378589 \|  \| \| -46,-8,54 \| 4.86 \| 0.000196099 \| 0.000386396 \|  \| \| 18,12,0 \| 4.857 \| 0.000206411 \| 0.000399621 \|  \| \| -50,-8,50 \| 4.852 \| 0.000206411 \| 0.000399621 \|  \| \| -30,-10,66 \| 4.851 \| 0.000211596 \| 0.000404951 \|  \| \| -12,4,0 \| 4.84 \| 0.000227094 \| 0.000429607 \|  \| \| -12,6,4 \| 4.837 \| 0.000227094 \| 0.000429607 \|  \| \| 20,4,64 \| 4.826 \| 0.00023222 \| 0.000429607 \|  \| \| 8,-22,-8 \| 4.802 \| 0.000258029 \| 0.000469528 \|  \| \| 44,18,2 \| 4.802 \| 0.000258029 \| 0.000469528 \|  \| \| 4,44,34 \| 4.796 \| 0.000263214 \| 0.00047636 \|  \| \| -20,-30,-2 \| 4.795 \| 0.00026834 \| 0.000483012 \|  \| \| -36,-4,56 \| 4.79 \| 0.000283837 \| 0.000505442 \|  \| \| -2,-24,-12 \| 4.783 \| 0.000299335 \| 0.000519926 \|  \| \| -34,-8,56 \| 4.774 \| 0.000304461 \| 0.000519926 \|  \| \| 60,10,18 \| 4.75 \| 0.000340641 \| 0.0005735 \|  \| \| -36,-6,60 \| 4.719 \| 0.000371575 \| 0.000618672 \|  \| \| -26,22,-2 \| 4.703 \| 0.000381887 \| 0.000629733 \|  \| \| 32,0,48 \| 4.697 \| 0.000387073 \| 0.00063184 \|  \| \| 6,30,32 \| 4.689 \| 0.00040257 \| 0.000650756 \|  \| \| 14,-26,0 \| 4.682 \| 0.000407696 \| 0.000655859 \|  \| \| -4,-20,54 \| 4.644 \| 0.000474811 \| 0.00074231 \|  \| \| 10,26,24 \| 4.644 \| 0.000474811 \| 0.00074231 \|  \| \| 6,24,24 \| 4.638 \| 0.000479937 \| 0.000743344 \|  \| \| -32,-10,46 \| 4.627 \| 0.000500619 \| 0.0007659 \|  \| \| -40,2,32 \| 4.625 \| 0.000500619 \| 0.0007659 \|  \| \| 58,2,14 \| 4.62 \| 0.000505745 \| 0.0007659 \|  \| \| -10,-30,68 \| 4.605 \| 0.000510931 \| 0.000769864 \|  \| \| 2,26,26 \| 4.596 \| 0.000526428 \| 0.000789642 \|  \| \| -36,4,8 \| 4.568 \| 0.000577986 \| 0.000851738 \|  \| \| 44,-4,50 \| 4.56 \| 0.000583172 \| 0.000851738 \|  \| \| 50,2,-6 \| 4.559 \| 0.000583172 \| 0.000851738 \|  \| \| -28,28,-4 \| 4.556 \| 0.000583172 \| 0.000851738 \|  \| \| 44,10,14 \| 4.505 \| 0.000681221 \| 0.000973591 \|  \| \| -4,-30,-18 \| 4.472 \| 0.00076896 \| 0.001089633 \|  \| \| -36,2,32 \| 4.468 \| 0.000779271 \| 0.001099565 \|  \| \| 52,0,14 \| 4.434 \| 0.000867009 \| 0.001183254 \|  \| \| 14,0,14 \| 4.428 \| 0.000887632 \| 0.001206455 \|  \| \| 14,-18,66 \| 4.424 \| 0.000898004 \| 0.001215591 \|  \| \| 50,-2,42 \| 4.415 \| 0.000913441 \| 0.001226516 \|  \| \| -36,24,12 \| 4.414 \| 0.000913441 \| 0.001226516 \|  \| \| 16,-4,16 \| 4.412 \| 0.000918627 \| 0.001228525 \|  \| \| -56,-32,48 \| 4.368 \| 0.001032174 \| 0.001358553 \|  \| \| 64,4,12 \| 4.357 \| 0.001068294 \| 0.001395066 \|  \| \| 52,2,6 \| 4.335 \| 0.001140535 \| 0.001466402 \|  \| \| -52,6,2 \| 4.321 \| 0.001197278 \| 0.001521731 \|  \| \| -20,6,60 \| 4.31 \| 0.001217961 \| 0.001530494 \|  \| \| -32,24,12 \| 4.3 \| 0.001279891 \| 0.00160227 \|  \| \| 22,-4,54 \| 4.298 \| 0.001285017 \| 0.001602662 \|  \| \| -18,22,-2 \| 4.29 \| 0.001316011 \| 0.001635193 \|  \| \| 52,14,2 \| 4.274 \| 0.001408875 \| 0.001731201 \|  \| \| -34,8,28 \| 4.271 \| 0.001429558 \| 0.001749999 \|  \| \| 50,4,0 \| 4.251 \| 0.00151211 \| 0.00183771 \|  \| \| 54,2,2 \| 4.224 \| 0.001661777 \| 0.001962311 \|  \| \| 8,0,2 \| 4.202 \| 0.00174433 \| 0.002030986 \|  \| \| 38,6,12 \| 4.193 \| 0.001775324 \| 0.002058125 \|  \| \| 10,-12,-16 \| 4.169 \| 0.00187856 \| 0.002157105 \|  \| \| 2,-24,28 \| 4.156 \| 0.0019508 \| 0.002208761 \|  \| \| -44,-40,56 \| 4.151 \| 0.001971424 \| 0.002208761 \|  \| \| -30,28,6 \| 4.15 \| 0.001971424 \| 0.002208761 \|  \| \| 14,-4,-12 \| 4.146 \| 0.001976609 \| 0.002208761 \|  \| \| 42,2,-16 \| 4.145 \| 0.001976609 \| 0.002208761 \|  \| \| 12,-40,54 \| 4.136 \| 0.002033353 \| 0.002249523 \|  \| \| 40,0,8 \| 4.121 \| 0.002126276 \| 0.002344536 \|  \| \| 38,0,-6 \| 4.118 \| 0.002141714 \| 0.002353765 \|  \| \| 60,12,26 \| 4.096 \| 0.002265573 \| 0.002481697 \|  \| \| -24,2,60 \| 4.07 \| 0.002420425 \| 0.002616888 \|  \| \| -4,24,16 \| 4.065 \| 0.00245136 \| 0.002641757 \|  \| \| -44,-20,60 \| 3.998 \| 0.003075838 \| 0.003220925 \|  \| \| -36,-6,10 \| 3.971 \| 0.003359675 \| 0.003474447 \|  \| \| -16,-12,60 \| 3.954 \| 0.003519654 \| 0.003617422 \|  \| \| 12,-32,42 \| 3.94 \| 0.003664196 \| 0.003720053 \|  \| \| -50,-24,56 \| 3.915 \| 0.003948033 \| 0.003959925 \|  \| | | | | | | | | | | | | | | | | | | |  |
| -28,-70,26 | 5.753 | | ~0 | | 0 | | 1854 | | | Left parieto-occipital transition zone | | | | | 0.359 | | | |  |
| \| -24,-72,26 \| 5.529 \| ~0 \| 0 \| Left precuneus \| \| --- \| --- \| --- \| --- \| --- \| \| -16,-76,38 \| 5.339 \| ~0 \| 0 \| Left parieto-occipital transition zone \| \| -18,-72,40 \| 5.306 \| ~0 \| 0 \| Left parieto-occipital transition zone \| \| -18,-72,10 \| 5.261 \| ~0 \| 0 \| Left striate area \| \| -14,-72,10 \| 5.162 \| ~0 \| 0 \| Left striate area \| \| -18,-66,36 \| 5.139 \| ~0 \| 0 \| Left parieto-occipital transition zone \| \| -30,-46,38 \| 5.129 \| ~0 \| 0 \| Left superior parietal lobule \| \| -10,-72,14 \| 5.123 \| ~0 \| 0 \| Left striate area \| \| -26,-50,44 \| 5.101 \| ~0 \| 0 \| Left superior parietal lobule \| \| -32,-48,44 \| 5.023 \| ~0 \| 0 \| Left supramarginal gyrus \| \| -28,-58,40 \| 4.973 \| 0.00016 \| 0.000343742 \|  \| \| -36,-78,6 \| 4.777 \| 0.000304 \| 0.000519926 \|  \| \| -24,-84,12 \| 4.776 \| 0.000304 \| 0.000519926 \|  \| \| -16,-72,32 \| 4.77 \| 0.000304 \| 0.000519926 \|  \| \| -22,-68,52 \| 4.705 \| 0.000382 \| 0.000629733 \|  \| \| -36,-78,10 \| 4.646 \| 0.000459 \| 0.000724887 \|  \| \| -26,-82,18 \| 4.61 \| 0.000506 \| 0.0007659 \|  \| \| -10,-82,28 \| 4.494 \| 0.000702 \| 0.000999 \|  \| \| -36,-86,14 \| 4.435 \| 0.000862 \| 0.001183254 \|  \| \| -30,-78,-6 \| 4.285 \| 0.001352 \| 0.001667467 \|  \| \| -30,-84,-2 \| 4.231 \| 0.001595 \| 0.00190371 \|  \| \| -16,-94,12 \| 4.226 \| 0.001641 \| 0.001944673 \|  \| \| -12,-96,8 \| 4.04 \| 0.00273 \| 0.002895191 \|  \| \| -20,-58,50 \| 4.019 \| 0.002916 \| 0.00306318 \|  \| \| -34,-38,48 \| 3.945 \| 0.003618 \| 0.003695687 \|  \| \| -28,-84,6 \| 3.944 \| 0.003633 \| 0.003699661 \|  \| \|  \|  \|  \|  \|  \| | | | | | | | | | | | | | | | | | | |  |
| 30,-48,38 | 5.352 | | ~0 | | 0 | | 1491 | | | Right supramarginal gyrus | | | | | | 0.185 | | |  |
| \| 30,-54,38 \| 5.207 \| ~0 \| 0 \| Right superior parietal lobule \| \| --- \| --- \| --- \| --- \| --- \| \| 34,-68,22 \| 5.06 \| ~0 \| 0 \| Right angular gyrus \| \| 20,-68,42 \| 5.016 \| ~0 \| 0 \| Right angular gyrus \| \| 22,-68,38 \| 4.986 \| 0.000155 \| 0.000343742 \|  \| \| 22,-80,26 \| 4.984 \| 0.000155 \| 0.000343742 \|  \| \| 22,-78,22 \| 4.957 \| 0.000165 \| 0.000352212 \|  \| \| 24,-68,2 \| 4.882 \| 0.000181 \| 0.00036309 \|  \| \| 24,-62,0 \| 4.867 \| 0.000191 \| 0.000378589 \|  \| \| 30,-80,14 \| 4.777 \| 0.000304 \| 0.000519926 \|  \| \| 26,-64,32 \| 4.745 \| 0.000356 \| 0.000595719 \|  \| \| 28,-72,26 \| 4.668 \| 0.000434 \| 0.000691493 \|  \| \| 20,-70,14 \| 4.666 \| 0.000434 \| 0.000691493 \|  \| \| 8,-66,10 \| 4.562 \| 0.000583 \| 0.000851738 \|  \| \| 22,-84,18 \| 4.433 \| 0.000867 \| 0.001183254 \|  \| \| 36,-50,52 \| 4.37 \| 0.001032 \| 0.001358553 \|  \| \| 12,-72,16 \| 4.354 \| 0.001079 \| 0.001398082 \|  \| \| 28,-60,-2 \| 4.325 \| 0.001192 \| 0.001520828 \|  \| \| 10,-78,12 \| 4.316 \| 0.001202 \| 0.001521924 \|  \| \| 34,-76,10 \| 4.316 \| 0.001208 \| 0.001523727 \|  \| \| 14,-64,4 \| 4.232 \| 0.001584 \| 0.001897381 \|  \| \| 38,-66,16 \| 4.219 \| 0.001677 \| 0.00197329 \|  \| \| 14,-90,20 \| 4.172 \| 0.001873 \| 0.002157105 \|  \| \| 28,-64,8 \| 4.071 \| 0.00242 \| 0.002616888 \|  \| \| 26,-64,24 \| 4.05 \| 0.002637 \| 0.00281449 \|  \| \| 38,-50,60 \| 3.987 \| 0.003169 \| 0.003287467 \|  \| | | | | | | | | | | | | | | | | | | |  |
| -30,-60,-10 | | 6.286 | | ~0 | | 0 | | 461 | Left hippocampus CA1 | | | | | | | | 0.587 | |  |
| \| -42,-56,-10 \| 5.687 \| ~0 \| 0 \| Left inferior temporal gyrus \| \| --- \| --- \| --- \| --- \| --- \| \| -38,-58,-8 \| 5.664 \| ~0 \| 0 \| Left inferior temporal gyrus \| \| -42,-66,4 \| 5.069 \| ~0 \| 0 \| Left middle temporal gyrus \| \| -18,-44,-8 \| 4.526 \| 0.000645 \| 0.000933848 \|  \| \| -18,-74,-6 \| 4.517 \| 0.000666 \| 0.00095594 \|  \| | | | | | | | | | | | | | | | | | | |  |
| 52,-34,36 | | 5.207 | | ~0 | | 0 | | 174 | | | | | | Right supramarginal gyrus | | | 0.654 | |  |
| 24,-46,-8 | | 5.28 | | ~0 | | 0 | | 88 | | | | | | Right fusiform gyrus | | | 0.620 | |  |
| 36,42,26 | | 4.457 | | 0.000794768 | | 0.001107355 | | 83 | | | | | | Right middle frontal gyrus | | | 0.184 | |  |
| 46,-56,-8 | | 4.827 | | 0.00023222 | | 0.000429607 | | 78 | | | | | | Right inferior temporal gyrus | | | 0.143 | |  |
| \| 40,-58,-8 \| 4.751 \| 0.000341 \| 0.0005735 \|  \| \| --- \| --- \| --- \| --- \| --- \| | | | | | | | | | | | | | | | | | | |  |
| -36,36,32 | | 5.056 | | ~0 | | 0 | | 70 | | | | | | Left middle frontal gyrus | | | 0.757 | |  |
| 32,-20,66 | | 4.829 | | 0.00023222 | | 0.000429607 | | 57 | | | | | | Right precentral gyrus | | | 0.670 | |  |
| -12,-24,36 | | 4.827 | | 0.00023222 | | 0.000429607 | | 44 | | | | | | Left posterior cingulate | | | 0.075 | |  |
| \| -6,-26,40 \| 4.147 \| 0.001971 \|  \| 0.002208761 \| \| --- \| --- \| --- \| --- \| --- \| | | | | | | | | | | | | | | | | | | |  |
| -32,-24,14 | | 4.524 | | 0.000660598 | | 0.000952291 | | 41 | | | | | | Left insula | | | 0.938 | |  |
| \| -34,-20,-2 \| 4.137 \| 0.002028 \|  \| 0.002249523 \| \| --- \| --- \| --- \| --- \| --- \| \| -36,-22,6 \| 4.046 \| 0.002678 \|  \| 0.002849118 \| | | | | | | | | | | | | | | | | | | |  |
| -36,-36,58 | | 4.381 | | 0.001006365 | | 0.001335138 | | 40 | | | | | | Left postcentral gyrus | | | 0.285 | |  |
| -50,-24,18 | | 4.336 | | 0.001140535 | | 0.001466402 | | 39 | | | | | | Left parietal operculum | | | 0.754 | |  |
| \| -54,-22,22 \| 4.242 \| 0.001553 \|  \| 0.001873626 \| \| --- \| --- \| --- \| --- \| --- \| | | | | | | | | | | | | | | | | | | |  |
| -14,-42,50 | | 4.215 | | 0.001687586 | | 0.001978754 | | 36 | | | | | | Left paracingulate | | | 0.074 | |  |
| \| -8,-40,52 \| 4.189 \| 0.00178 \|  \| 0.002058125 \| \| --- \| --- \| --- \| --- \| --- \| | | | | | | | | | | | | | | | | | | |  |
| 56,-16,38 | | 4.237 | | 0.001558542 | | 0.001873626 | | 20 | | | | | | Right postcentral gyrus | | | 0.407 | |  |
| -26,48,-10 | | 4.617 | | 0.000505745 | | 0.0007659 | | 14 | | | | | | Left intermediate orbital gyrus | | | 0.650 | |  |
| 4,-40,56 | | 4.055 | | 0.002580404 | | 0.002762941 | | 14 | | | | | | Right paracingulate | | | 0.071 | |  |
| \| 8,-40,54 \| 3.992 \| 0.003259016 \|  \| Right paracingulate \| \| --- \| --- \| --- \| --- \| --- \| \| 6,-44,54 \| 3.963 \| 0.003559904 \|  \| Right paracingulate \| | | | | | | | | | | | | | | | | | | |  |
| 2,-48,-16 | | 3.947 | | 0.003586769 | | 0.003675059 | | 12 | | | | | Right cerebellum anterior lobe culmen** | | | | 0.058 | |  |

MNI (Montreal Neurological Institute), SDM-z (Signed Differential Mapping z-score), FDR (false discovery rate), *nearest grey matter structure, **Talairach client

1. Anticipation lose-vs-neutral maps only deactivation all peaks

| **Peak MNI coordinate** | **SDM-z** | **P** | **FDR** | **Voxels** | **Anatomical Description** | **Egger’s test p** |
| --- | --- | --- | --- | --- | --- | --- |
| 8,64,16 | -3.4 | ~0 | 0 | 5037 | Right superior frontopolar gyrus | 0.435 |
| \| 12,60,24 \| -2.838 \| ~0 \| 0 \| \| --- \| --- \| --- \| --- \| \| 10,56,24 \| -2.79 \| ~0 \| 0 \| \| -4,62,18 \| -2.494 \| ~0 \| 0 \| \| -2,34,0 \| -2.42 \| ~0 \| 0 \| \| -26,26,48 \| -2.389 \| ~0 \| 0 \| \| 20,62,24 \| -2.381 \| ~0 \| 0 \| \| -8,60,8 \| -2.33 \| ~0 \| 0 \| \| 2,54,-14 \| -2.266 \| ~0 \| 0 \| \| 18,30,50 \| -2.235 \| ~0 \| 0 \| \| -12,60,24 \| -2.234 \| ~0 \| 0 \| \| -2,54,16 \| -2.206 \| ~0 \| 0 \| \| -36,16,44 \| -2.151 \| 0.000005186 \| 1.56E-05 \| \| -42,12,52 \| -2.12 \| 0.000005186 \| 1.56E-05 \| \| -14,38,44 \| -2.034 \| 0.000030994 \| 9.22E-05 \| \| -8,60,30 \| -2.024 \| 0.00003612 \| 0.000105508 \| \| -20,38,38 \| -1.973 \| 0.000041306 \| 0.000117563 \| \| 16,40,42 \| -1.937 \| 0.000051618 \| 0.000140892 \| \| 0,22,-8 \| -1.933 \| 0.000051618 \| 0.000140892 \| \| 30,30,52 \| -1.912 \| 0.000051618 \| 0.000140892 \| \| -6,56,0 \| -1.863 \| 0.000072241 \| 0.000182244 \| \| -6,48,-4 \| -1.863 \| 0.000072241 \| 0.000182244 \| \| -16,50,30 \| -1.831 \| 0.000072241 \| 0.000182244 \| \| -8,42,-6 \| -1.825 \| 0.000077426 \| 0.000192409 \| \| -18,46,30 \| -1.775 \| 0.00009805 \| 0.000240078 \| \| 38,24,52 \| -1.668 \| 0.000159979 \| 0.000343742 \| \| -16,54,30 \| -1.657 \| 0.000159979 \| 0.000343742 \| \| 42,18,48 \| -1.611 \| 0.000175476 \| 0.000360701 \| \| -10,24,56 \| -1.581 \| 0.000206411 \| 0.000399621 \| \| -6,58,34 \| -1.299 \| 0.000799954 \| 0.001109936 \| \| -34,10,58 \| -1.225 \| 0.001047671 \| 0.001373521 \| \| -20,60,6 \| -1.121 \| 0.001548231 \| 0.001873626 \| \| -12,44,24 \| -1.105 \| 0.001620471 \| 0.001927203 \| \| 30,18,60 \| -1.088 \| 0.001728892 \| 0.002020074 \| \| 26,20,60 \| -1.047 \| 0.001955926 \| 0.002208761 \| \| -26,30,56 \| -0.984 \| 0.002353311 \| 0.002569353 \| \| -12,56,38 \| -0.977 \| 0.002399802 \| 0.002611549 \| \| -20,58,12 \| -0.932 \| 0.002828121 \| 0.002989728 \| | | | | | | |
| -52,-66,32 | -3.609 | ~0 | 0 | 2191 | Left superior temporal gyrus | 0.199 |
| \| -54,-62,40 \| -3.588 \| ~0 \| 0 \| \| --- \| --- \| --- \| --- \| \| -36,-74,44 \| -3.146 \| ~0 \| 0 \| \| -42,-72,44 \| -3.082 \| ~0 \| 0 \| \| -34,-78,42 \| -2.919 \| ~0 \| 0 \| \| -48,-58,26 \| -2.778 \| ~0 \| 0 \| \| -38,-68,42 \| -2.739 \| ~0 \| 0 \| \| -62,-54,22 \| -1.982 \| 0.000041306 \| 0.000117563 \| \| -42,-84,24 \| -1.897 \| 0.000061929 \| 0.000164979 \| \| -62,-48,30 \| -1.304 \| 0.000789583 \| 0.001107355 \| | | | | | | |
| -14,-52,32 | -2.661 | ~0 | 0 | 1974 | Left precuneus | 0.456 |
| \| -12,-48,28 \| -2.59 \| ~0 \| 0 \| \| --- \| --- \| --- \| --- \| \| -6,-60,28 \| -2.569 \| ~0 \| 0 \| \| -4,-50,10 \| -2.353 \| ~0 \| 0 \| \| -12,-56,26 \| -2.343 \| ~0 \| 0 \| \| 4,-60,30 \| -2.331 \| ~0 \| 0 \| \| 2,-44,10 \| -1.795 \| 0.000092924 \| 0.000229213 \| | | | | | | |
| 52,-68,34 | -4.054 | ~0 | 0 | 1609 | Right angular gyrus | 0.790 |
| \| 58,-62,30 \| -3.977 \| ~0 \| 0 \| \| --- \| --- \| --- \| --- \| \| 52,-64,30 \| -3.606 \| ~0 \| 0 \| \| 60,-58,30 \| -3.57 \| ~0 \| 0 \| \| 62,-56,16 \| -3.062 \| ~0 \| 0 \| \| 64,-52,14 \| -3.011 \| ~0 \| 0 \| \| 60,-60,12 \| -2.733 \| ~0 \| 0 \| \| 68,-40,2 \| -2.003 \| 0.00003612 \| 0.000105508 \| \| 66,-48,-2 \| -1.891 \| 0.000061929 \| 0.000164979 \| \| 66,-44,4 \| -1.883 \| 0.000061929 \| 0.000164979 \| \| 66,-32,0 \| -1.842 \| 0.000072241 \| 0.000182244 \| \| 70,-30,0 \| -1.819 \| 0.000077426 \| 0.000192409 \| | | | | | | |
| -60,-12,-16 | -2.375 | ~0 | 0 | 1105 | Left middle temporal gyrus | 0.333 |
| \| -64,-18,-18 \| -2.179 \| ~0 \| 0 \| \| --- \| --- \| --- \| --- \| \| -54,-10,-32 \| -2.133 \| 0.000005186 \| 1.56E-05 \| \| -54,-6,-26 \| -1.943 \| 0.000051618 \| 0.000140892 \| \| -48,12,-36 \| -1.377 \| 0.000588357 \| 0.000855558 \| \| -54,4,-32 \| -1.3 \| 0.000794768 \| 0.001107355 \| \| -64,-40,-8 \| -1.292 \| 0.000820577 \| 0.001133826 \| \| -60,-40,-8 \| -1.256 \| 0.000970244 \| 0.001292365 \| \| -48,6,-38 \| -1.162 \| 0.001321197 \| 0.001635534 \| \| -60,-6,-6 \| -1.049 \| 0.001935303 \| 0.002208761 \| \| -42,16,-40 \| -0.787 \| 0.004417658 \| 0.004417658 \| | | | | | | |
| 58,0,-18 | -2.752 | ~0 | 0 | 577 | Right middle temporal gyrus | 0.986 |
| \| 66,-12,0 \| -1.872 \| 0.000072241 \| 0.000182244 \| \| --- \| --- \| --- \| --- \| \| 54,-6,-30 \| -1.759 \| 0.000103235 \| 0.000247318 \| \| 52,10,-32 \| -1.692 \| 0.00013417 \| 0.000312438 \| \| 54,12,-24 \| -1.618 \| 0.000175476 \| 0.000360701 \| \| 42,18,-40 \| -1.577 \| 0.000211596 \| 0.000404951 \| \| 44,0,-34 \| -1.569 \| 0.000237405 \| 0.000436773 \| \| 48,6,-42 \| -1.496 \| 0.000340641 \| 0.0005735 \| \| 50,4,-32 \| -1.424 \| 0.000479937 \| 0.000743344 \| \| 64,-4,-2 \| -1.038 \| 0.002028227 \| 0.002249523 \| | | | | | | |
| -44,38,-18 | -2.156 | 0.000005186 | 1.56E-05 | 413 | Left inferior frontal gyrus orbital part* | 0.862 |
| \| -54,24,8 \| -1.997 \| 0.000041306 \| 0.000117563 \| \| --- \| --- \| --- \| --- \| \| -48,28,4 \| -1.951 \| 0.000046432 \| 0.000131033 \| \| -40,32,-16 \| -1.692 \| 0.00013417 \| 0.000312438 \| \| -48,30,-2 \| -1.668 \| 0.000159979 \| 0.000343742 \| \| -48,34,-12 \| -1.548 \| 0.000283837 \| 0.000505442 \| \| -38,44,-16 \| -0.896 \| 0.003153265 \| 0.003281366 \| | | | | | | |
| -16,-16,-26 | -2.75 | ~0 | 0 | 337 | Left entorhinal cortex | 0.862 |
| \| -22,-10,-22 \| -2.343 \| ~0 \| 0 \| \| --- \| --- \| --- \| --- \| \| -22,2,-24 \| -1.697 \| 0.00013417 \| 0.000312438 \| | | | | | | |
| 2,-58,66 | -1.88 | 0.000072241 | 0.000182244 | 302 | Right paracingulate gyrus | **0.006** |
| \| 4,-62,64 \| -1.855 \| 0.000072241 \| 0.000182244 \| \| --- \| --- \| --- \| --- \| \| 4,-82,44 \| -1.766 \| 0.000103235 \| 0.000247318 \| \| 0,-76,52 \| -1.536 \| 0.000294149 \| 0.000519926 \| \| 2,-90,30 \| -1.528 \| 0.000304461 \| 0.000519926 \| \| -2,-74,58 \| -1.457 \| 0.00040257 \| 0.000650756 \| \| 4,-88,34 \| -1.38 \| 0.000577986 \| 0.000851738 \| \| -6,-68,64 \| -0.847 \| 0.003684819 \| 0.003729619 \| | | | | | | |
| 20,-26,-26 | -1.436 | 0.000459313 | 0.000724887 | 124 | Right cerebellum anterior lobe culmen** | 0.929 |
| \| 24,-20,-26 \| -1.215 \| 0.001078606 \| 0.001398082 \| \| --- \| --- \| --- \| --- \| \| 18,-18,-26 \| -1.2 \| 0.001156032 \| 0.00148061 \| \| 20,-20,-20 \| -0.953 \| 0.002570093 \| 0.002760777 \| \| 12,-12,-24 \| -0.928 \| 0.002848744 \| 0.003001999 \| | | | | | | |
| 34,38,-12 | -1.766 | 0.000103235 | 0.000247318 | 96 | Right lateral orbital gyrus | 0.255 |
| \| 42,38,-18 \| -1.648 \| 0.00017029 \| 0.000358902 \| \| --- \| --- \| --- \| --- \| | | | | | | |
| -30,-32,-20 | -1.601 | 0.000175476 | 0.000360701 | 39 | Left fusiform gyrus | 0.903 |
| 4,-28,74 | -1.139 | 0.001434684 | 0.001749999 | 14 | Right precentral gyrus | **0.026** |
| \| 2,-38,74 \| -0.834 \| 0.003818989 \| 0.003853707 \| \| --- \| --- \| --- \| --- \| \| -2,-40,72 \| -0.828 \| 0.00389123 \| 0.003914742 \| | | | | | | |
| 56,28,18 | -1.716 | 0.000123858 | 0.000294605 | 12 | Right inferior frontal gyrus opercular part * | 0.201 |
| 20,-36,12 | -1.473 | 0.000387073 | 0.00063184 | 10 | Right stria terminalis | 0.957 |

MNI (Montreal Neurological Institute), SDM-z (Signed Differential Mapping z-score), FDR (false discovery rate), *nearest grey matter structure, **Talairach client

**Supplementary Table 6: Between Group Linear Comparison anticipation win-vs-neutral minus anticipation lose-vs-neutral**

| **MNI coordinate** | **SDM-z** | **P** | **FDR** | **Voxels** | **Description** |
| --- | --- | --- | --- | --- | --- |
| -6,38,18 | 2.923 | 0.003034532 | 0.004327869 | 8 | Left anterior cingulate |
| -44,38,-10 | -2.776 | 0.000676095 | 0.004327869 | 36 | Left inferior frontal gyrus orbital part |

MNI (Montreal Neurological Institute), SDM-z (Signed Differential Mapping z-score), FDR (false discovery rate)

**Supplementary Table 7: Hetereogeneity QH Stats Converted to SDM z-scores**

1. Anticipation win-vs-neutral

| Peak MNI coordinate | SDM-z | Voxels | Anatomical Description |
| --- | --- | --- | --- |
| -30,-92,-4 | 8.371 | 3305 | Left occipital gyrus |
| 34,-88,-2 | 7.99 | 3090 | Right occipital gyrus |
| 10,4,-6 | 7.171 | 1000 | Right nucleus basalis |
| 30,20,-6 | 6.915 | 611 | Right posterior orbital gyrus |
| -38,20,0 | 6.439 | 192 | Left inferior frontal gyrus orbital part |
| -42,-22,54 | 5.703 | 150 | Left postcentral gyrus |
| -10,10,-6 | 5.784 | 95 | Left nucleus accumbens |
| -14,-10,66 | 5.793 | 87 | Left superior frontal gyrus lateral part |
| -4,-54,64 | 6.154 | 62 | Left superior parietal lobule |
| -30,-58,52 | 5.376 | 54 | Left superior parietal lobule |
| -54,6,12 | 5.658 | 38 | Left inferior frontal gyrus opercular part |
| 50,4,34 | 5.295 | 38 | Right precentral gyrus |
| -52,8,-6 | 5.39 | 34 | Left superior temporal gyrus |
| -12,-72,-4 | 5.782 | 33 | Left occipital gyrus |
| -22,-24,-8 | 5.869 | 32 | Left lateral geniculate nucleus |
| 32,-12,-6 | 5.992 | 24 | Right limitans claustrum |
| 2,-66,0 | 5.726 | 23 | Right cerebellum anterior lobe culmen |
| -10,-16,42 | 5.334 | 17 | Left posterior cingulate |
| -10,-12,56 | 5.351 | 16 | Left superior frontal gyrus lateral part |
| 2,-12,68 | 5.392 | 13 | Right superior frontal gyrus lateral part |
| -40,-64,24 | 5.295 | 13 | Left angular gyrus |
| 60,8,22 | 5.295 | 13 | Right precentral gyrus |
| -50,-26,54 | 5.574 | 11 | Left postcentral gyrus |
| -60,-32,32 | 5.167 | 11 | Left parietal operculum |

MNI (Montreal Neurological Institute), SDM-z (Signed Differential Mapping z-score)

1. Anticipation lose-vs-neutral

| Peak MNI coordinate | SDM-z | Voxels | Anatomical Description |
| --- | --- | --- | --- |
| -42,-22,48 | 7.021 | 1171 | Left postcentral gyrus |
| -6,-8,50 | 6.745 | 593 | Left paracentral lobule |
| 2,-52,-20 | 6.807 | 400 | Right cerebellum anterior lobe culmen* |
| 10,6,2 | 6.221 | 192 | Right fundus of the caudate |
| 38,-8,54 | 6.057 | 117 | Right precentral gyrus* |
| -4,-24,-6 | 6.704 | 87 | Left red nucleus |
| -8,6,0 | 5.715 | 79 | Left nucleus accumbens |
| -24,-96,-8 | 5.654 | 37 | Left occipital gyrus |
| 6,-48,36 | 5.544 | 35 | Right posterior cingulat |
| -18,-60,16 | 5.702 | 15 | Left occipital gyrus |
| 32,24,2 | 5.295 | 14 | Right inferior frontal gyrus opercular part |
| 56,-60,24 | 5.295 | 11 | Right superior temporal gyrus |
| 0,40,-14 | 5.368 | 11 | Left straight gyrus |
| -8,-82,4 | 5.295 | 10 | Left occipital gyrus |

MNI (Montreal Neurological Institute), SDM-z (Signed Differential Mapping z-score)

**Supplementary Figure 2: Binarised thresholded overlay map of heterogeneity and mean map activation and deactivation**


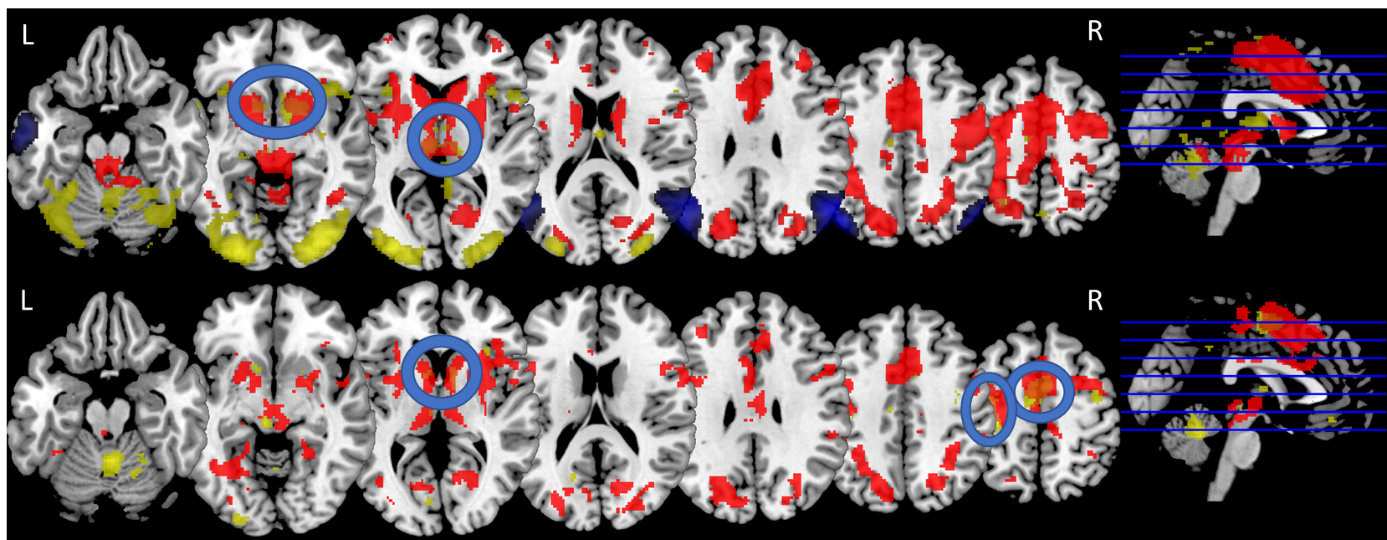
Top row anticipation win-vs-neutral; bottom anticipation lose-vs-neutral; heterogeneity is yellow, activation is red, deactivation is blue, blue circles indicate visual overlap

**Supplementary Figure 3: Binarised Jacknife density maps with mean maps of activation and deactivation separated out to enhance visual inspection**

1. Anticipation win-vs-neutral


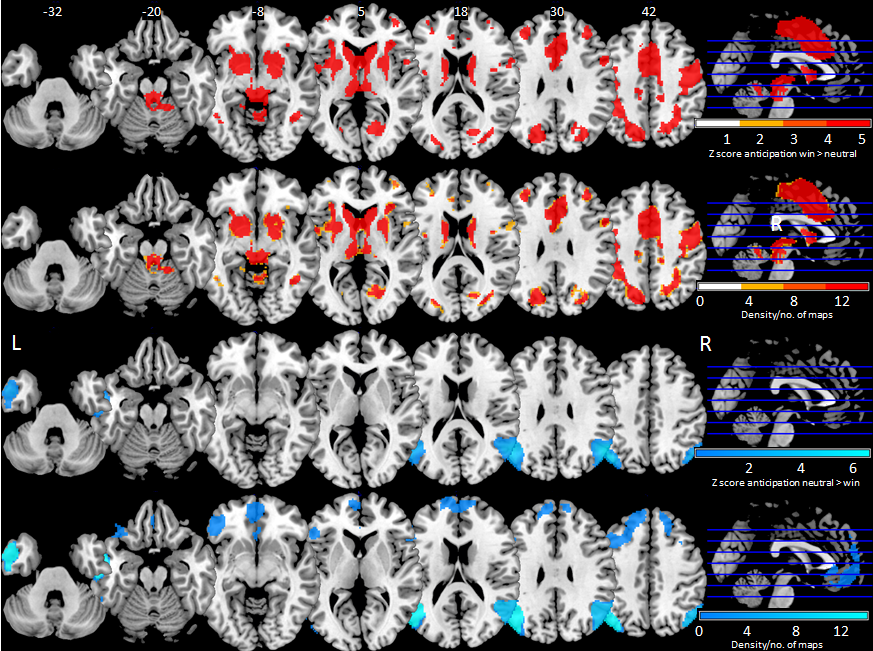


Descending from the top row: mean map activation, Jacknife density map activation, mean map deactivation, Jacknife density map deactivation

1. Anticipation lose-vs-neutral


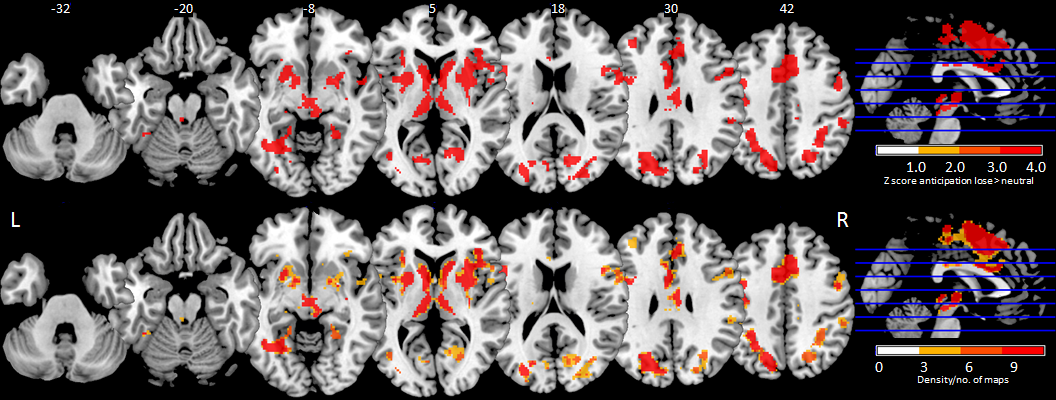


Top row mean map activation, bottom row Jacknife density map deactivation

**Supplementary Figure 4: Meta-regression for placebo effect with mean map heterogeneity overlayed**

**
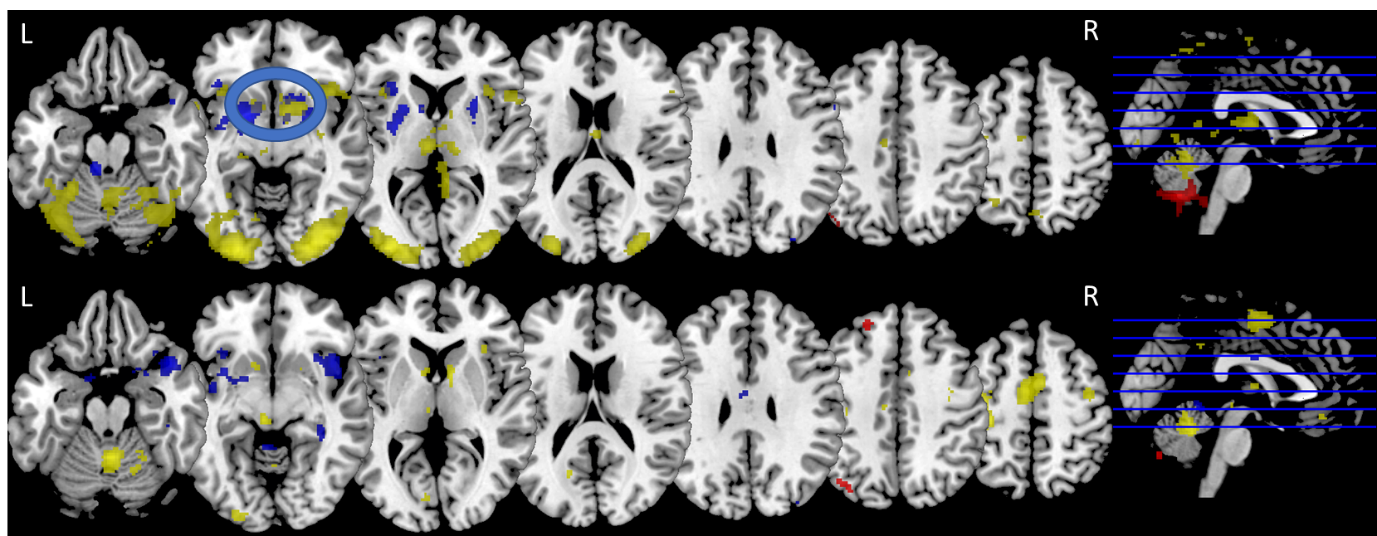
**

Top row- anticipation win-vs-neutral; bottom anticipation lose-vs-neutral; heterogeneity in yellow, activation red, deactivation blue, blue circles indicate visual overlap

**Supplementary Table 8: Meta-regression for Placebo Effect**

1. Anticipation win-vs-neutral

| Peak MNI coordinate | SDM-z | Voxels | Anatomical description |
| --- | --- | --- | --- |
| 0,-60,-42 | 3.649 | 2614 | Left cerebellum posterior lobe** |
| -46,-72,40 | 2.738 | 28 | Left angular gyrus |
| 28,24,48 | 2.322 | 22 | Right middle frontal gyrus |
| -16,2,-8 | -4.112 | 991 | Left substriatal terminal island (encompassing ventral striatum) |
| 24,0,-8 | -3.421 | 212 | Right putamen |
| 12,12,-12 | -3.855 | 152 | Right posteromedial orbital lobule |
| -36,26,-12 | -3.152 | 151 | Left inferior frontal gyrus orbital part |
| -8,-34,-18 | -3.963 | 80 | Left cerebellum anterior lobe** |
| 32,-84,30 | -3.351 | 65 | Right occipital gyrus |
| -56,-2,12 | -3.064 | 65 | Left precentral gyrus |
| 46,12,-18 | -3.091 | 30 | Right middle temporal gyrus |
| 60,8,34 | -3.200 | 15 | Right inferior frontal gyrus opercular part* |
| 48,6,-30 | -3.003 | 15 | Right inferior temporal gyrus |
| -44,-20,22 | -2.882 | 15 | Left frontal operculum |
| -4,-4,28 | -3.114 | 10 | Left posterior cingulate gyrus |

MNI (Montreal Neurological Institute), SDM-z (Signed Differential Mapping z-score), *nearest grey matter structure, **Talairach client

1. Anticipation lose-vs-neutral

| Peak MNI coordinate | SDM-z | Voxels | Anatomical description |
| --- | --- | --- | --- |
| 16,-64,-52 | 3.268 | 123 | Right cerebellum posterior lobe* |
| -22,36,46 | 3.125 | 109 | Left superior frontal gyrus lateral part |
| -6,-72,-40 | 3.341 | 57 | Left cerebellum posterior lobe* |
| -12,-58,-56 | 2.759 | 43 | Left cerebellum posterior lobe* |
| -36,-72,44 | 2.879 | 33 | Left angular gyrus |
| -14,60,12 | 2.943 | 30 | Left middle frontopolar gyrus |
| -8,56,28 | 2.912 | 13 | Left superior frontal gyrus medial part |
| -20,56,24 | 2.672 | 11 | Left superior frontal gyrus lateral part |
| -50,-60,42 | 2.667 | 10 | Left supramarginal gyrus |

MNI (Montreal Neurological Institute), SDM-z (Signed Differential Mapping z-score), *Talairach client

**Supplementary Figure 5: Meta-regression for Field Strength with Mean Map Heterogeneity Overlay**

**
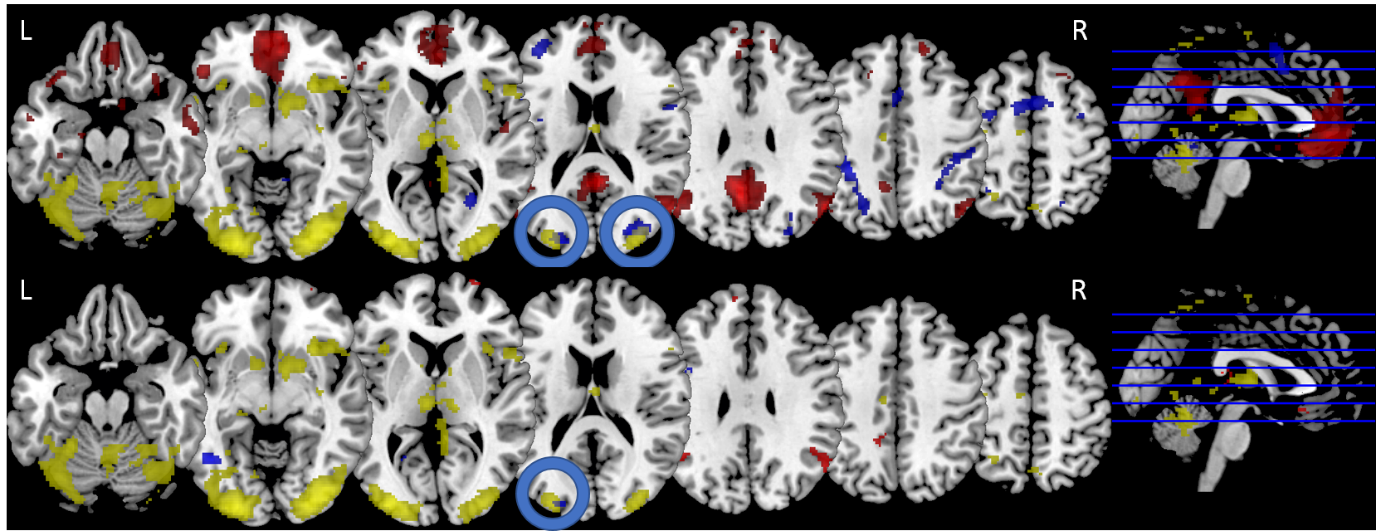
**

Top row- anticipation win-vs-neutral; bottom anticipation lose-vs-neutral; heterogeneity in yellow, activation red, deactivation blue, blue circles indicate visual overlap

**Supplementary Table 9: Meta-regression for Field Strength**

1. Anticipation win-vs-anticipation neutral

| Peak MNI coordinate | SDM-z | Voxels | Anatomical description |
| --- | --- | --- | --- |
| -56,-56,36 | 2.533 | 105 | Left supramarginal gyrus |
| -14,-46,38 | 2.837 | 101 | Left posterior cingulate gyrus |
| 56,-58,34 | 2.424 | 93 | Right superior temporal gyrus |
| 24,68,0 | 2.544 | 32 | Right inferior frontopolar gyrus |
| 0,-30,14 | 2.55 | 20 | Left thalamus |
| -2,26,-12 | 2.126 | 19 | Left straight gyrus |
| -6,54,34 | 2.184 | 18 | Left superior frontal gyrus, lateral part |
| -48,-54,-12 | -5.115 | 127 | Left inferior temporal gyrus |
| -22,-50,0 | -4.309 | 56 | Left lingual gyrus |
| -22,-88,16 | -4.485 | 34 | Left occipital gyri |
| -40,4,34 | -3.747 | 30 | Left precentral gyrus |
| -20,-38,-28 | -4.214 | 13 | Left cerebellum anterior lobe culmen |
| -6,-68,8 | -3.511 | 12 | Left striate area |

MNI (Montreal Neurological Institute), SDM-z (Signed Differential Mapping z-score)

1. Anticipation lose-vs-anticipation neutral

| Peak MNI coordinate | SDM-z | Voxels | Anatomical description |
| --- | --- | --- | --- |
| -4,34,-14 | 4.495 | 2513 | Left straight gyrus |
| -4,-56,30 | 5.278 | 1405 | Left posterior cingulate gyrus |
| -52,-66,32 | 4.104 | 661 | Left angular gyrus |
| -66,-34,-6 | 3.917 | 616 | Left middle temporal gyrus |
| 54,-64,28 | 5.038 | 507 | Right superior temporal gyrus |
| 48,0,-32 | 3.305 | 297 | Right inferior temporal gyrus |
| -48,30,-10 | 3.515 | 256 | Left inferior frontal gyrus, orbital part |
| 18,46,42 | 3.325 | 122 | Right superior frontal gyrus, lateral part |
| 36,18,-32 | 2.709 | 85 | Right inferior temporopolar region |
| 8,8,-16 | 2.544 | 56 | Right anterior olfactory nucleus |
| -30,-38,-16 | 3.241 | 52 | Left fusiform gyrus |
| -42,18,48 | 3.078 | 50 | Left middle frontal gyrus |
| 34,26,-18 | 2.603 | 52 | Right posterior orbital gyrus |
| -18,-14,-24 | 2.983 | 30 | Left entorhinal cortex |
| 24,26,58 | 2.514 | 30 | Right superior frontal gyrus, lateral part |
| 50,26,-4 | 2.532 | 21 | Right inferior frontal gyrus, triangular part |
| -6,42,30 | 2.284 | 21 | Left superior frontal gyrus, medial part |
| 50,-10,4 | 2.553 | 20 | Right anterior transverse temporal gyrus |
| -32,-14,-24 | 2.355 | 17 | Left parahippocampal gyrus |
| 58,-24,-22 | 2.32 | 16 | Right inferior temporal gyrus |
| -18,30,44 | 2.427 | 14 | Left superior frontal gyrus, lateral part |
| -48,-32,52 | -5.857 | 1642 | Left supramarginal gyrus |
| 8,6,62 | -5.31 | 613 | Right superior frontal gyrus, medial part |
| 28,-50,46 | -4.397 | 418 | Right superior parietal lobule |
| -52,4,14 | -5.091 | 372 | Right precentral gyrus |
| 28,-84,22 | -5.509 | 344 | Right parietal-occipital transition zone |
| -38,44,22 | -4.45 | 196 | Left middle frontal gyrus |
| -22,-90,18 | -5.226 | 100 | Left occipital gyri |
| 24,-60,6 | -4.524 | 50 | Right striate area |
| -42,-2,50 | -4.27 | 46 | Left middle frontal gyrus |
| 38,36,34 | -4.364 | 42 | Right middle frontal gyrus |
| 60,8,14 | -4.408 | 44 | Right precentral gyrus |
| -16,-18,72 | -5.059 | 25 | Left precentral gyrus |
| 8,-6,70 | -4.806 | 19 | Right superior frontal gyrus, lateral part |
| 34,0,54 | -3.864 | 22 | Right middle frontal gyrus |
| -30,-76,24 | -3.828 | 15 | Left angular gyrus |
| 36,50,24 | -3.725 | 15 | Right middle frontal gyrus |
| -62,-16,16 | -3.506 | 14 | Left reticular thalamic nucleus |
| 0,-50,-12 | -3.42 | 13 | Left cerebellum, anterior lobe culmen |
| 62,-40,36 | -3.762 | 11 | Right superior temporal gyrus |

MNI (Montreal Neurological Institute), SDM-z (Signed Differential Mapping z-score)

**Supplementary References 2: all papers originally included in omnibus analysis**

Balodis, I. M., Kober, H., Worhunsky, P. D., Stevens, M. C., Pearlson, G. D., & Potenza, M. N. (2012). Diminished frontostriatal activity during processing of monetary rewards and losses in pathological gambling. *Biol Psychiatry, 71*(8), 749-757, doi:10.1016/j.biopsych.2012.01.006.

Beck, A., Schlagenhauf, F., Wüstenberg, T., Hein, J., Kienast, T., Kahnt, T., et al. (2009). Ventral striatal activation during reward anticipation correlates with impulsivity in alcoholics. *Biol Psychiatry, 66*(8), 734-742, doi:10.1016/j.biopsych.2009.04.035.

Bustamante, J. C., Barros-Loscertales, A., Costumero, V., Fuentes-Claramonte, P., Rosell-Negre, P., Ventura-Campos, N., et al. (2014). Abstinence duration modulates striatal functioning during monetary reward processing in cocaine patients. *Addict Biol, 19*(5), 885-894, doi:10.1111/adb.12041.

Choi, J. S., Shin, Y. C., Jung, W. H., Jang, J. H., Kang, D. H., Choi, C. H., et al. (2012). Altered brain activity during reward anticipation in pathological gambling and obsessive-compulsive disorder. *PLoS One, 7*(9), e45938, doi:10.1371/journal.pone.0045938.

Costumero, V., Barros-Loscertales, A., Bustamante, J. C., Ventura-Campos, N., Fuentes, P., & Avila, C. (2013). Reward sensitivity modulates connectivity among reward brain areas during processing of anticipatory reward cues. *Eur J Neurosci, 38*(3), 2399-2407, doi:10.1111/ejn.12234.

da Silva Alves, F., Schmitz, N., Figee, M., Abeling, N., Hasler, G., van der Meer, J., et al. (2011). Dopaminergic modulation of the human reward system: a placebo-controlled dopamine depletion fMRI study. *Journal of Psychopharmacology, 25*(4), 538-549.

Damiano, C. R., Aloi, J., Dunlap, K., Burrus, C. J., Mosner, M. G., Kozink, R. V., et al. (2014). Association between the oxytocin receptor (OXTR) gene and mesolimbic responses to rewards. *Mol Autism, 5*(1), 7, doi:10.1186/2040-2392-5-7.

de Greck, M., Scheidt, L., Bölter, A. F., Frommer, J., Ulrich, C., Stockum, E., et al. (2011). Multimodal psychodynamic psychotherapy induces normalization of reward related activity in somatoform disorder. *World Journal of Biological Psychiatry, 12*(4), 296-308.

de Leeuw, M., Kahn, R. S., & Vink, M. (2015). Fronto-striatal dysfunction during reward processing in unaffected siblings of schizophrenia patients. *Schizophr Bull, 41*(1), 94-103, doi:10.1093/schbul/sbu153.

Enzi, B., Edel, M. A., Lissek, S., Peters, S., Hoffmann, R., Nicolas, V., et al. (2012). Altered ventral striatal activation during reward and punishment processing in premanifest Huntington's disease: a functional magnetic resonance study. *Exp Neurol, 235*(1), 256-264, doi:10.1016/j.expneurol.2012.02.003.

Funayama, T., Ikeda, Y., Tateno, A., Takahashi, H., Okubo, Y., Fukayama, H., et al. (2014). Modafinil augments brain activation associated with reward anticipation in the nucleus accumbens. *Psychopharmacology (Berl), 231*(16), 3217-3228, doi:10.1007/s00213-014-3499-0.

Jansma, J. M., van Hell, H. H., Vanderschuren, L. J., Bossong, M. G., Jager, G., Kahn, R. S., et al. (2013). THC reduces the anticipatory nucleus accumbens response to reward in subjects with a nicotine addiction. *Transl Psychiatry, 3*, e234, doi:10.1038/tp.2013.6.

Kappel, V., Lorenz, R. C., Streifling, M., Renneberg, B., Lehmkuhl, U., Strohle, A., et al. (2014). Effect of brain structure and function on reward anticipation in children and adults with attention deficit hyperactivity disorder combined subtype. *Soc Cogn Affect Neurosci*, doi:10.1093/scan/nsu135.

Kaufmann, C., Beucke, J. C., Preusse, F., Endrass, T., Schlagenhauf, F., Heinz, A., et al. (2013). Medial prefrontal brain activation to anticipated reward and loss in obsessive-compulsive disorder. *Neuroimage Clin, 2*, 212-220, doi:10.1016/j.nicl.2013.01.005.

Kirk, U., Brown, K. W., & Downar, J. (2014). Adaptive neural reward processing during anticipation and receipt of monetary rewards in mindfulness meditators. *Soc Cogn Affect Neurosci*, doi:10.1093/scan/nsu112.

Knutson, B., Adams, C. M., Fong, G. W., & Hommer, D. (2001a). Anticipation of increasing monetary reward selectively recruits nucleus accumbens. *J Neurosci, 21*(16), RC159.

Knutson, B., Bhanji, J. P., Cooney, R. E., Atlas, L. Y., & Gotlib, I. H. (2008). Neural responses to monetary incentives in major depression. *Biol Psychiatry, 63*(7), 686-692, doi:10.1016/j.biopsych.2007.07.023.

Knutson, B., Fong, G. W., Adams, C. M., Varner, J. L., & Hommer, D. (2001b). Dissociation of reward anticipation and outcome with event-related fMRI. *Neuroreport, 12*(17), 3683-3687.

Knutson, B., Fong, G. W., Bennett, S. M., Adams, C. M., & Hommer, D. (2003). A region of mesial prefrontal cortex tracks monetarily rewarding outcomes: characterization with rapid event-related fMRI. *Neuroimage, 18*(2), 263-272.

Li, Y., Sescousse, G., & Dreher, J.-C. (2014). Endogenous cortisol levels are associated with an imbalanced striatal sensitivity to monetary versus non-monetary cues in pathological gamblers. *Frontiers in behavioral neuroscience, 8*.

Nestor, L., Hester, R., & Garavan, H. (2010). Increased ventral striatal BOLD activity during non-drug reward anticipation in cannabis users. *Neuroimage, 49*(1), 1133-1143, doi:10.1016/j.neuroimage.2009.07.022.

Ossewaarde, L., Qin, S., Van Marle, H. J., van Wingen, G. A., Fernández, G., & Hermans, E. J. (2011). Stress-induced reduction in reward-related prefrontal cortex function. *Neuroimage, 55*(1), 345-352.

Ossewaarde, L., Verkes, R. J., Hermans, E. J., Kooijman, S. C., Urner, M., Tendolkar, I., et al. (2011). Two-week administration of the combined serotonin-noradrenaline reuptake inhibitor duloxetine augments functioning of mesolimbic incentive processing circuits. *Biol Psychiatry, 70*(6), 568-574, doi:10.1016/j.biopsych.2011.03.041.

Pecina, M., Martinez-Jauand, M., Love, T., Heffernan, J., Montoya, P., Hodgkinson, C., et al. (2014). Valence-specific effects of BDNF Val66Met polymorphism on dopaminergic stress and reward processing in humans. *J Neurosci, 34*(17), 5874-5881, doi:10.1523/jneurosci.2152-13.2014.

Pfabigan, D. M., Seidel, E. M., Sladky, R., Hahn, A., Paul, K., Grahl, A., et al. (2014). P300 amplitude variation is related to ventral striatum BOLD response during gain and loss anticipation: an EEG and fMRI experiment. *Neuroimage, 96*, 12-21, doi:10.1016/j.neuroimage.2014.03.077.

Romanczuk-Seiferth, N., Koehler, S., Dreesen, C., Wustenberg, T., & Heinz, A. (2014). Pathological gambling and alcohol dependence: neural disturbances in reward and loss avoidance processing. *Addict Biol*, doi:10.1111/adb.12144.

Saji, K., Ikeda, Y., Kim, W., Shingai, Y., Tateno, A., Takahashi, H., et al. (2013). Acute NK(1) receptor antagonist administration affects reward incentive anticipation processing in healthy volunteers. *Int J Neuropsychopharmacol, 16*(7), 1461-1471, doi:10.1017/s1461145712001678.

Spreckelmeyer, K. N., Krach, S., Kohls, G., Rademacher, L., Irmak, A., Konrad, K., et al. (2009). Anticipation of monetary and social reward differently activates mesolimbic brain structures in men and women. *Soc Cogn Affect Neurosci, 4*(2), 158-165, doi:10.1093/scan/nsn051.

Treadway, M. T., Buckholtz, J. W., & Zald, D. H. (2013). Perceived stress predicts altered reward and loss feedback processing in medial prefrontal cortex. *Front Hum Neurosci, 7*, 180, doi:10.3389/fnhum.2013.00180.

Vaidya, J. G., Knutson, B., O'Leary, D. S., Block, R. I., & Magnotta, V. (2013). Neural sensitivity to absolute and relative anticipated reward in adolescents. *PLoS One, 8*(3), e58708, doi:10.1371/journal.pone.0058708.

van Hell, H. H., Jager, G., Bossong, M. G., Brouwer, A., Jansma, J. M., Zuurman, L., et al. (2012). Involvement of the endocannabinoid system in reward processing in the human brain. *Psychopharmacology (Berl), 219*(4), 981-990, doi:10.1007/s00213-011-2428-8.

Weiland, B. J., Heitzeg, M. M., Zald, D., Cummiford, C., Love, T., Zucker, R. A., et al. (2014). Relationship between impulsivity, prefrontal anticipatory activation, and striatal dopamine release during rewarded task performance. *Psychiatry Res, 223*(3), 244-252, doi:10.1016/j.pscychresns.2014.05.015.

Wu, C. C., Samanez-Larkin, G. R., Katovich, K., & Knutson, B. (2014). Affective traits link to reliable neural markers of incentive anticipation. *Neuroimage, 84*, 279-289, doi:10.1016/j.neuroimage.2013.08.055.
